# The C3/465 glycan hole cluster in BG505 HIV-1 envelope is the major neutralizing target involved in preventing mucosal SHIV infection

**DOI:** 10.1371/journal.ppat.1009257

**Published:** 2021-02-08

**Authors:** Tysheena P. Charles, Samantha L. Burton, Prabhu S. Arunachalam, Christopher A. Cottrell, Leigh M. Sewall, Venkata S. Bollimpelli, Sailaja Gangadhara, Antu K. Dey, Andrew B. Ward, George M. Shaw, Eric Hunter, Rama R. Amara, Bali Pulendran, Marit J. van Gils, Cynthia A. Derdeyn

**Affiliations:** 1 Emory Vaccine Center, Emory University, Atlanta, Georgia, United States of America; 2 Yerkes National Primate Research Center, Emory University, Atlanta, Georgia, United States of America; 3 Departments of Pathology, and Microbiology and Immunology, Institute for Immunity, Transplantation and Infection, Stanford University School of Medicine, Stanford, California, United States of America; 4 Department of Integrative Structural and Computational Biology, The Scripps Research Institute, La Jolla, California, United States of America; 5 International AIDS Vaccine Initiative, New York, New York, United States of America; 6 Department of Medicine, University of Pennsylvania, Philadelphia, Pennsylvania, United States of America; 7 Department of Pathology and Laboratory Medicine, Emory University, Atlanta, Georgia, United States of America; 8 Department of Microbiology and Immunology, Emory University, Atlanta, Georgia, United States of America; 9 Department of Medical Microbiology, Amsterdam UMC, University of Amsterdam, Amsterdam, The Netherlands; Northwestern University Feinberg School of Medicine, UNITED STATES

## Abstract

Stabilized HIV-1 envelope (Env) trimers elicit tier 2 autologous neutralizing antibody (nAb) responses in immunized animals. We previously demonstrated that BG505 SOSIP.664.T332N gp140 (BG505 SOSIP) immunization of rhesus macaques (RM) provided robust protection against autologous intra-vaginal simian-human immunodeficiency virus (SHIV) challenge that was predicted by high serum nAb titers. Here, we show that nAb in these protected RM targeted a glycan hole proximal to residue 465 in gp120 in all cases. nAb also targeted another glycan hole at residues 241/289 and an epitope in V1 at varying frequencies. Non-neutralizing antibodies directed at N611-shielded epitopes in gp41 were also present but were more prevalent in RM with low nAb titers. Longitudinal analysis demonstrated that nAb broadened in some RM during sequential immunization but remained focused in others, the latter being associated with increases in nAb titer. Thirty-eight monoclonal antibodies (mAbs) isolated from a protected RM with an exceptionally high serum neutralization titer bound to the trimer in ELISA, and four of the mAbs potently neutralized the BG505 Env pseudovirus (PV) and SHIV. The four neutralizing mAbs were clonally related and targeted the 465 glycan hole to varying degrees, mimicking the serum. The data demonstrate that the C3/465 glycan hole cluster was the dominant neutralization target in high titer protected RM, despite other co-circulating neutralizing and non-neutralizing specificities. The isolation of a neutralizing mAb family argues that clonotype expansion occurred during BG505 SOSIP immunization, leading to high titer, protective nAb and setting a desirable benchmark for HIV vaccines.

## Author summary

Despite much effort, an effective vaccine against HIV/AIDS has not been developed. There are many obstacles that continue to undermine this effort, which is focused largely on inducing neutralizing antibodies that can protect against infection. We conducted a pre-clinical vaccine study in nonhuman primates that utilized a trimeric HIV-1 envelope vaccine to induce neutralizing antibodies. We found that all animals that developed very high levels of neutralizing antibodies were protected against infection. When we mapped the neutralizing antibody responses using viral mutants, we found that they mainly targeted one exposed region of the HIV-1 envelope protein that is unique to the strain we used in the vaccine. By isolating individual antibodies from a protected animal with a very high level of neutralizing antibodies, we found that an expanded antibody family was responsible for the neutralization. Our findings demonstrate that neutralizing antibodies targeting mainly one exposed region on the envelope trimer vaccine were associated with protection, and that this could be achieved by a single antibody family that develops and expands during immunization.

## Introduction

Successful vaccines have been developed against many viral pathogens by inducing nAb responses that protect against infection [1]. Achieving robust protective efficacy has proven especially difficult for HIV vaccines due to the high genetic diversity and conformational complexity of HIV-1 Env, the sole target for nAb. Human vaccine trials have had limited success in terms of protecting against HIV-1 acquisition. None have elicited antibodies that can neutralize patient-derived tier 2 variants that exhibit nAb resistance and circulate throughout the pandemic [2]. The exposure of irrelevant epitopes on monomeric and poorly trimeric Env vaccine immunogens is an obstacle for eliciting tier 2 neutralizing antibodies; however, the development of stabilized, native-like gp140 trimers has provided a major advance [3–5]. Native-like SOSIP trimers have elicited antibodies with tier 2 autologous neutralizing capacity in small animal models and in RM [6–9]. A recent study provided evidence that high serum nAb 50% inhibitory dilution (ID_50_) titers elicited by BG505 SOSIP were associated with protection against repeated, low dose intra-rectal SHIV.BG505 challenges [10]. However, the *post-hoc* design and small numbers of RM in that study did not support evaluation of protective efficacy. We recently conducted a pre-clinical efficacy trial in which RM were immunized with BG505 SOSIP in 3M-052 adjuvant alone, or in combination with three heterologous viral vectors (HVV) expressing SIVmac239 Gag (vesicular stomatitis virus (VSV)-Gag, vaccinia virus (VV)-Gag and adenovirus type 5 (Ad5)-Gag). The HVV did not express Env and were included to test whether anti-Gag T cell responses also contributed to protection. Our study also included a control group that received only the 3M-052 adjuvant. Immunization resulted in robust and significant protection against repeated, low dose intra-vaginal SHIV.BG505 challenges in both vaccination arms, compared to the controls [9]. A serum ID_50_ titer greater than 1:319 at the antibody peak, two weeks after final immunization, was completely predictive of protection, prompting us to examine in greater detail the specificities and characteristics of nAb associated with preventing acquisition.

Previous small animal and RM studies have demonstrated that antibodies elicited by immunization with stabilized trimers, including BG505 SOSIP, mediate autologous neutralization by targeting highly immunogenic regions devoid of asparagine (N)-linked glycosylation, or ‘glycan holes’ [7,8,11–15]. The location of glycan holes in BG505 Env is unique, which restricts neutralizing activity to the autologous strain [7]. In BG505 SOSIP immunized rabbits, nAb recognized a non-glycosylated region of gp120 centered on positions 241/289 [7,12]. In that study, mutations that insert a glycan motif at position 241 or 289 in the parental BG505 Env rendered the PV more resistant to neutralization by immune sera. Another autologous neutralization target was identified in immunized rabbits and RM located near the gp120 C3 and V5 regions and this can be blocked by introduction of a glycan motif at 465 [12,14,15]. A third glycan-mediated neutralizing epitope has been identified in immunized rabbits and RM near residues 133 and 136 in the V1 hypervariable domain [12,14–16]. This V1-targeted neutralization can be blocked by introducing a C-terminal glycan shift and elongating the V1 hypervariable domain (see S2B Fig) [16]. Non-neutralizing antibodies that recognize epitopes in gp41 that are proximal to the trimer base have also been described [7,17,18]. While these antibodies do not neutralize the parental BG505 Env PV, removal of a glycan motif through an N611A substitution significantly enhances neutralization.

Here we expand on nAb-mediated protection against intra-vaginal challenge by defining the targets recognized in 12 immunized RM. A panel of BG505 Env mutants [12,19] was used to map nAb on the day of challenge and at preceding time points in a subset of animals. This approach, combined with negative stain electron microscopy polyclonal epitope mapping (nsEMPEM), revealed that BG505 SOSIP-elicited nAb targeted one or more sites in the 465 glycan hole cluster in all RM, often in combination with an array of other neutralizing and non-neutralizing specificities. Longitudinal mapping during immunization indicated that N611 antibodies arose prior to nAb in some RM, and that when nAb remained focused on one region, such as the 465 glycan hole, this drove an increase in nAb titer. To further inform our analysis, we isolated 48 mAbs from a protected RM that developed the highest autologous serum nAb titer. Thirty-eight mAbs bound to the BG505 SOSIP in ELISA, and four of those mAbs were clonally related, and potently neutralized BG505 Env PV and BG505.SHIV *in vitro* despite moderate levels of somatic hypermutation, and were blocked to varying degrees by closing the 465 glycan hole. Overall, the data provides new insight into the dominant immunogenicity of the C3/465 glycan hole cluster on BG505 SOSIP and that this contributed to the development of high nAb titers associated with robust protection [9].

## Results

### Protected RM have high serum nAb titers against BG505 Env PV and BG505 SHIV

We previously demonstrated in our preclinical efficacy study that BG505 SOSIP immunization of RM provided statistically significant protection against ten intra-vaginal challenges with BG505 SHIV [9]. Briefly, two groups of 15 RM received four subcutaneous immunizations with BG505 SOSIP in 3M-052 adjuvant (S1A Fig). One of the groups also received SIVmac239 Gag-expressing HVV to boost T cell responses (S1A Fig). A third group of 15 unimmunized RM received only 3M-052 adjuvant. Significant protection was observed in the SOSIP (p = 0.0006) and HVV + SOSIP (p<0.0001) vaccination groups compared to the control group [9].

**Fig 1:**
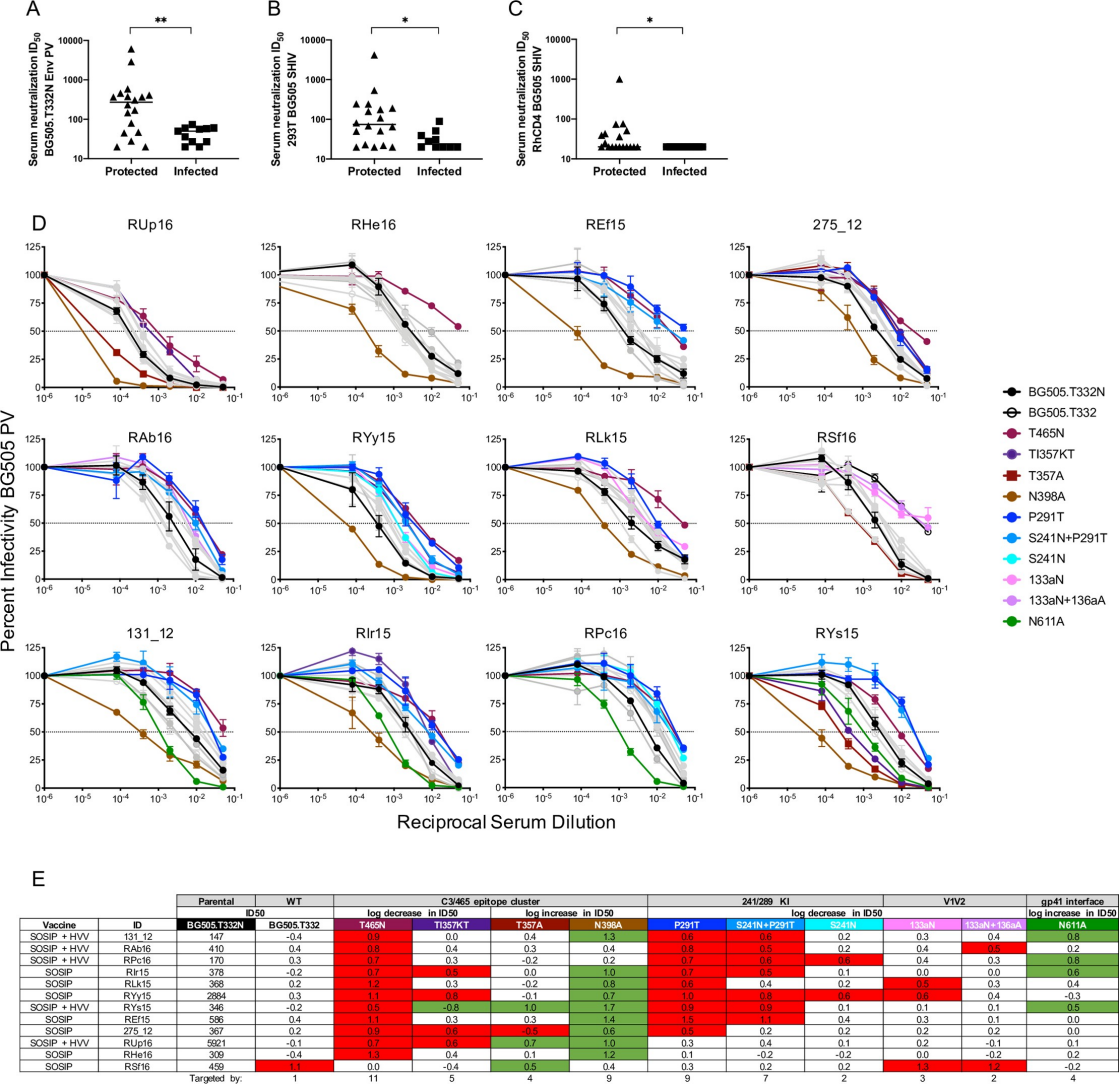
Mapping of serum neutralizing activity in high titer, protected RM using BG505 Env PV mutants. Serum neutralizing activity on the day of challenge (week 84) was evaluated using the TZM-bl assay (**A—E**) and ID_50_ titers were calculated using GraphPad prism. Neutralization activity for protected vs. infected RM against (**A)** BG505 Env PV (p = 0.0057), and BG505.SHIV produced in (**B)** 293T cells (p = 0.0120) and **(C)** rhesus CD4^+^ T cells (p = 0.0187) is shown. In each case, nAb ID_50_ titer was higher in protected RM using the Mann-Whitney test (*p<0.05, **p<0.01). For (**A)** through (**C),** the horizontal bar represents the median nAb ID_50_ titer for each group. Day of challenge serum neutralizing activity was also mapped using mutant BG505 Env PVs. Infectivity curves are depicted in (**D)** with the key indicating the color for each BG505 mutant PV as follows. The parental BG505 Env PV with T332N is shown with black closed symbols, while the BG505 Env PV with T332 is shown with black open symbols. Mutant PVs that had a reduction or increase in ID_50_ titer of 0.5log10 or greater compared to the parental BG505 are represented in color and symbol as listed in the key. The gray infectivity curves represent mutant PVs that did not reach the pre-defined threshold of a 0.5log10 fold increase or decrease in ID_50_ compared to the parental BG505 Env. The reciprocal of the serum dilution is plotted on the x axis on a log10 scale and the percent of viral infectivity is plotted on the y axis relative to the virus only control at 100%. Each serum-PV combination was run in duplicate, and each assay was repeated independently at least twice. The error bars represent the SEM for each data point. (**E)** the relative change in ID_50_ titer for each BG505 mutant PV compared to the parental BG505 PV is shown. The second row represents whether a log fold decrease or increase in ID_50_ titer is expected. The third row identifies each mutant. The ID_50_ titer against the parental BG505 containing T332N is shown in the third column and is the comparator for all mutants. The number of RM that targeted a particular region/mutant is shown in the last row. Mutants with a substantial decrease (red) or increase (green) in ID_50_ are highlighted, and correspond to the neutralization curves shown in (**D)**.

BG505 SOSIP specific serum IgG levels were assessed in an enzyme-linked immunosorbent assay (ELISA) (S1B and S1C Fig). At challenge, there was no significant difference in BG505 SOSIP-specific IgG levels between the two groups (S1B Fig) or protected vs. infected RM (S1C Fig). Serum neutralization was initially assessed against a PV expressing HIV-1 BG505.W6M.Env.C2 modified to contain the N332 glycan (T332N), herein referred to as the BG505 Env PV, to match the SOSIP immunogen. On day of challenge, BG505 SOSIP immunized RM exhibited a 2log10 range of autologous nAb ID_50_ titers, varying from undetectable (1:20) to 1:6068 (Fig 1A). There was no significant difference in nAb ID_50_ titers between HVV + SOSIP vs. SOSIP immunized RM (Mann-Whitney, p>0.05) (S1D Fig). In contrast, the nAb ID_50_ titers against BG505 Env PV were significantly higher in protected RM compared to those that became infected (Mann-Whitney, p = 0.0057) (Fig 1A).

Serum nAb titers against BG505 SHIV, produced by transfection of 293T cells with the infectious molecular clone or by growth in RM CD4^+^ T cells, were also significantly higher in protected vs infected RM (Mann-Whitney, p = 0.0120 and p = 0.0187, respectively) (Fig 1B and 1C). We selected 12 protected RM, five from the HVV + SOSIP group and seven from the SOSIP group, that exhibited nAb ID_50_ titers greater than 1:100 (S1D Fig) for nAb mapping studies to provide insight into the nature of protection. The threshold in nAb ID_50_ titer of at least 1:100 was imposed to provide a sufficient window with which to detect a reduction.

### Neutralizing antibodies in protected, high titer RM primarily target the C3/465 glycan hole cluster

To map serum nAb activity elicited by BG505 SOSIP, we used an initial panel of previously described mutant BG505 Env PVs with strategically altered glycan-encoding motifs [12,17,20]. This panel included two mutants that shift a glycan in V1 (133aN and 133aN + 136aA) (the “a” refers to insertion of an N or Alanine (A) residue into the BG505 V1 loop), one that closes a glycan hole near C3/V5 (T465N), three that close a glycan hole near 241/289 (P291T, S241N, P291T + S241N), and one that removes a glycan to expose epitopes in gp41 near the trimer base (N611A) (S2 Fig). The first six BG505 Env mutants described above were designed to decrease susceptibility to neutralization by blocking a glycan hole in gp120; however, the N611A mutant in gp41 is expected to become more susceptible to neutralization than the parent if proximal epitopes were targeted. Because the BG505 SOSIP immunogen we used was modified to encode the N332 glycan that is important for recognition by one class of bnAbs, we also evaluated the neutralization sensitivity of BG505 Env PV that lacked this glycan, which is representative of the wildtype patient derived BG505 Env.

Serum collected on day of challenge was used for the cross-sectional nAb mapping studies. Neutralization of parental (T332N), wildtype (T332) (WT), and mutant BG505 Env PVs (all in the T332N background) is shown in Fig 1D and 1E. To identify dominant epitopes targeted by nAbs in each animal, a threshold was set for a fold reduction in ID_50_ of 0.5log10 or greater compared to parental BG505 Env PV. Serum from all but one RM (RSf16) neutralized BG505 Env PV containing N332 and T332 with similar ID_50_ values, and a significant correlation was observed between the two PVs (r = 0.7932, p <0.0001) (S1E Fig). Notably, RSf16 serum neutralized the T332 PV with 1log10 lower potency than the T332N PV, suggesting that this glycan could participate in formation of a nAb epitope. Serum neutralization was independent of the N332 glycan in the remaining 11 RM, even though this glycan is present on the immunogen.

All but one of the RM had reduced neutralization capacity against the T465N mutant, establishing that epitopes proximal to the 465 glycan hole are most frequently targeted in these high titer, protected RM (Fig 1E). In two RM, RUp16 and RHe16, neutralization was directed predominantly at the 465 glycan hole, as only T465N PV reached the threshold for resistance (Fig 1D and 1E). Nine of the 465 glycan hole targeting RM had reduced neutralizing activity against one or more mutants in the P291T/S241N series that close the 241/289 glycan hole (Fig 1D and 1E). Given that the T465N mutant PV exhibited substantial resistance to neutralization by the majority of serum, we evaluated its susceptibility to neutralization by five HIV-1 bnAbs that target epitopes located in the V1V2 apex (PGDM1400, PGT145), the V3/mannose patch (PGT121), and the CD4 binding site (b12, VRC01), in parallel with the parental Env PV. The BG505 T465N PV exhibited a pattern of neutralization susceptibility that was indistinguishable from the wildtype Env (S3 Fig) and consistent with previous studies [4]. Therefore, the frequently reduced neutralization of T465N by immunized RM sera was not due to a pan-resistance phenotype.

To gain further insight into the specificities of serum nAbs targeting the 465 glycan hole, we performed additional mapping experiments using three mutants that have been used to define recognition of sites within the BG505 C3/465 epitope cluster [12,19]. These mutants shift (TI357KT) or remove (T357A to remove N355; N398A knock out) glycans in this region (S2 Fig) [12]. Of the 11 RM that had reduced serum neutralization against T465N, four were also reduced by the TI357KT glycan shift. Interestingly, one RM neutralized the TI357KT mutant PV better than the parental BG505 (Fig 1D and 1E). Removal of the proximal N355 glycan via the T357A substitution also had mixed effects. This mutation increased susceptibility to serum nAb by two 465 glycan hole targeting RM but impeded neutralization by a third (Fig 1D and 1E). Interestingly, the only RM to target V1 and N332 for neutralization was also affected by T357A. The removal of the N398 glycan motif caused dramatic increases in neutralization susceptibility to serum from nine 465 glycan hole targeting RMs (Fig 1D and 1E). Finally, serum nAb from two 465 glycan hole targeting RM did not reach the threshold for reduction or increase against the TI357KT, T357A, or N398A mutant PVs (Fig 1D and 1E). Consistent with previous studies, nAb in only four animals, RAb16, RYy15, RLk15, and RSf16 also targeted V1 (Fig 1D and 1E) [4,5]. Taken together, the results demonstrate that nAb from all RM was altered against at least one mutant from within the C3/465 glycan hole cluster, providing strong evidence that this exposed region contains dominant nAb targets in high titer, protected RM. However, there was substantial heterogeneity in recognition of nAb epitopes within this region. It is important to note that the 465 glycan hole is unique to BG505. While this region is highly immunogenic and associated with protection in our study, it is highly variable across HIV-1 variants and commonly occluded by one or more N-linked glycan motifs (S4 Fig). Thus, BG505 SOSIP elicited nAb targeted to the 465 glycan hole region appear to be heterogenous and likely restricted to strain-specificity. It is notable that none of the mutant BG505 PVs were completely resistant to serum neutralization (Fig 1D). In most experiments, neutralization of the mutant PVs at the highest serum concentration was 50 to 75%, suggesting there are diverse populations of antibodies contributing to neutralization, that the epitopes themselves are complex, or that there is substantial heterogeneity in glycosylation of the Env PVs. As an example, the S241N mutant was less efficient than P291T at reducing neutralization directed at the 241/289 glycan hole, indicating that perhaps the N241 site was not fully occupied.

We also assessed the effect of removing the N611 glycan motif on neutralization susceptibility using a 0.5log10 fold increase in ID_50_ titer as the threshold. Four high titer, protected RM, 131_12, RIr15, RPc16, and RYs15 showed substantial increases in neutralization of the N611A mutant compared to parental BG505 PV, in addition to bona fide nAb against the glycan holes (Fig 1D and 1E). Due to the increased susceptibility of N611A PV, we also tested RM serum that had ID_50_ titers below 1:100, thereby producing a near complete N611A data set (S5 Fig). This analysis revealed several intriguing but preliminary findings. Significantly lower levels of N611 targeting were observed in the protected RM compared to those that became infected (p = 0.05, Mann-Whitney). In fact, the high titer protected group was statistically different from low titer RM stratified as protected (p = 0.006) or infected (p = 0.004) using the previously defined nAb ID_50_ predictive threshold of 1:319 [9] (Kruskal-Wallis with Dunn’s correction). These observations suggest that the N611 glycan hole “nAbs” are not useful and could potentially interfere with protective neutralizing capacity. Since N611 is proximal to the gp41 fusion peptide, we also examined immunized RM sera for binding to a His-tagged version of the BG505 fusion peptide by ELISA. Serum from only two out of the 30 immunized RM had any detectable binding, which was much lower than the bnAb VRC34 that was used as a control (S6 Fig) [21]. The inability to detect these antibodies in serum could indicate that they are induced at low frequency by BG505 SOSIP immunization and/or that they require glycans or other gp120 contacts to bind to the peptide.

### nsEMPEM reveals co-circulating antibodies that bind to multiple sites on the SOSIP

**Fig 2:**
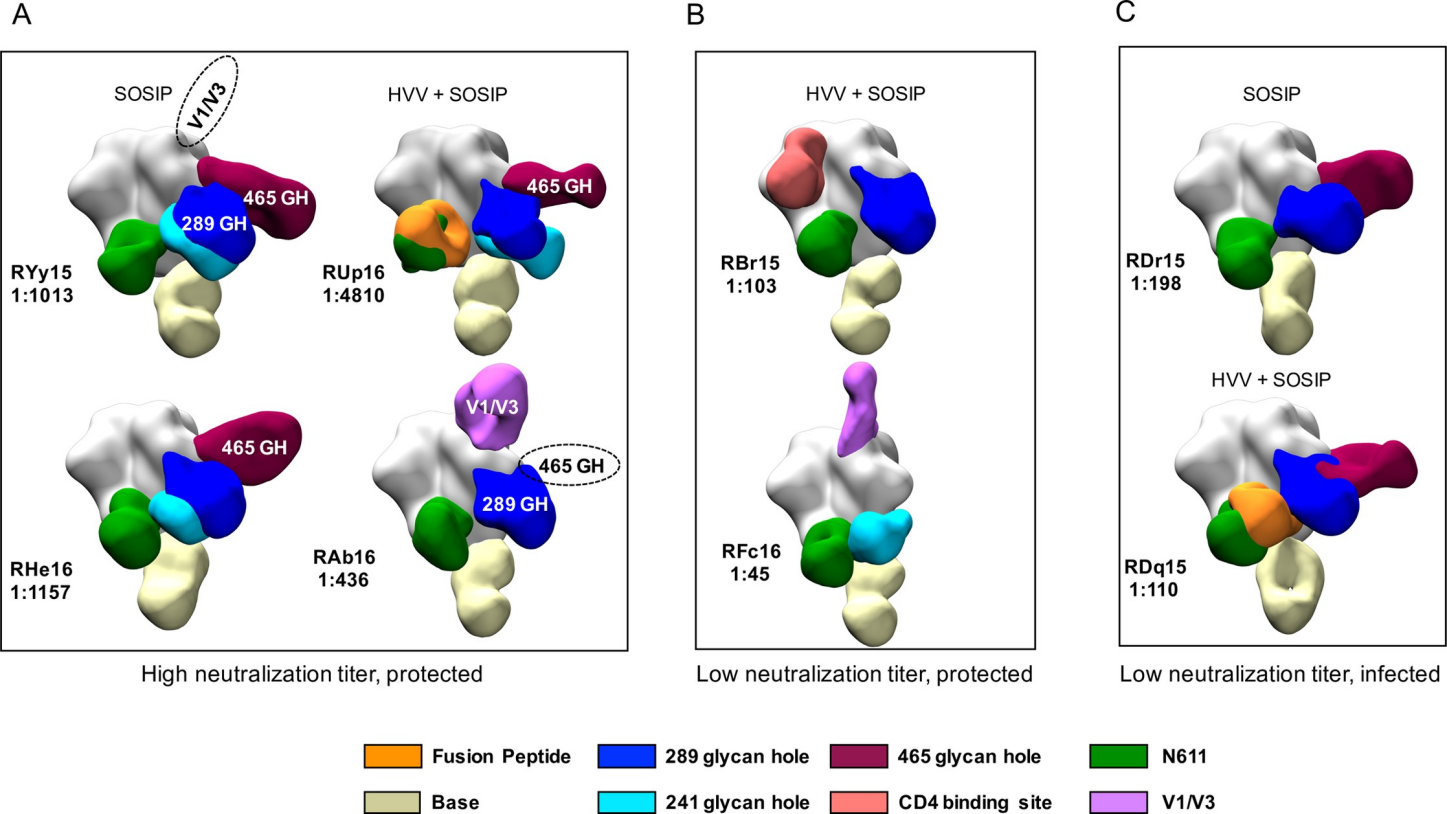
Visualization of antibody binding to BG505 SOSIP. nsEMPEM of BG505 SOSIP in complex with Fabs derived from serum IgG at week 82 is shown. Eight RM were selected for analysis based on their vaccine group, neutralization ID_50_ titer, and challenge outcome, as indicated in (**A—C)**. The code for each RM and the neutralization ID_50_ titer against BG505 Env PV is shown. Previously defined epitopes on BG505 SOSIP are coded by color in the key. Dashed ovals indicate antibody specificities detected by serum nAb mapping but not nsEMPEM; white text indicates antibody specificities that were detected by serum nAb mapping and nsEMPEM.

Regardless of nAb ID_50_ titer, all RM developed high levels of serum anti-BG505 SOSIP IgG (S1B and S1C Fig). To gain a more complete picture of the specificities of these co-circulating antibodies and determine whether there was a pattern associated with protection, fragments antigen binding (Fabs) derived from serum IgG from eight animals two weeks after the final SOSIP immunization were complexed with BG505 SOSIP and visualized using nsEMPEM [17]. RM from both vaccination groups that included protected animals with high or low nAb ID_50_ titer and infected animals with low nAb ID_50_ titer, measured against the parental BG505 Env PV, were analyzed. We visualized and compared circulating SOSIP-binding IgG specificities to gain additional insight into how antibodies may have impacted the different outcomes (Fig 2). The circulating antibody population was polyclonal in each RM with binding to eight epitope regions: the CD4 binding site, the trimer base, the 241, 289, and 465 glycan holes, the gp41 fusion peptide, N611, and V1/V3. All RM developed antibodies against the trimer base, the N611 region, and the 241 and 289 glycan holes. In RHe16, only antibodies against the 465 glycan hole contributed strongly to serum neutralization (Fig 1D and 1E), even though antibodies against the 241 and 289 glycan hole epitopes were also present (Fig 2A). Interestingly, for RAb16 and RYy15, the serum nAb mapping analysis picked up V1 and the 465 glycan hole as being dominant specificities (Fig 1D and 1E), but these were not observed by nsEMPEM (Fig 2A). Conversely, N611 targeting antibodies were detected by nsEMPEM (Fig 2A), but neutralization of the N611A mutant was not detected in any of the high titer, protected RM analyzed by nsEMPEM (Fig 1D and 1E). We also included two HVV + SOSIP immunized RM that had nAb ID_50_ titers below the predictive threshold (1:319) yet were still protected (Fig 2B) [9]. Antibodies in these RM also recognized multiple specificities that overlapped with high titer RM but both lacked binding to the 465 glycan hole. RM with titers less than 1:319 that became infected (Fig 2C) had profiles of binding antibodies similar to the protected RM, including the 465 and 289 glycan holes. Overall, there was no association between number of targeted sites or recognition profile that was predictive of nAb ID_50_ titer or protection.

### Longitudinal serum nAb mapping shows shifting or focusing of targets with subsequent immunizations

**Fig 3:**
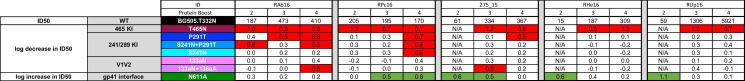
Longitudinal serum nAb mapping. Neutralization activity against the BG505 parental and mutant PVs was assessed using serum collected from five RM at two to four weeks after the second, third, and fourth SOSIP immunizations (weeks 26, 42/44, or 84) using the TZM-bl assay. Neutralization in serum from two RM (RAb16 and RPc16) was mapped using a panel of seven mutants at all timepoints because the nAb ID_50_ titers against the parental BG505 Env PV exceeded 1:100. The remaining RM had titers against the parental BG505 PV below 1:100 at week 26 and were only mapped against N611A at this timepoint. The RM is indicated in the first row, and the SOSIP immunizations, 2, 3, and 4, are indicated in the second row. The nAb titer for the parent BG505 PV is shown in the third row, with the log fold reduction or increase for each mutant PV in the rows below. Each mutant is indicated in the third column, and whether there is an expected decrease or increase in ID_50_ in the first column. Red shading indicates a log fold reduction of 0.5log10 of greater; green shading indicates a log fold increase of 05.log10 or greater. N/A indicates not analyzed due to low titer.

Our results demonstrate that binding and neutralizing antibodies capable of recognizing multiple BG505 SOSIP targets are consistently present after the fourth and final protein boost. We next compared the nAb specificities in serum collected at two weeks following the second and third BG505 SOSIP immunizations to what was present on the day of challenge. Two RM, RAb16 and RPc16, had nAb ID_50_ titers greater than 1:100 after the second and third BG505 SOSIP immunizations, which facilitated mapping with multiple mutant PVs in the panel. After two BG505 SOSIP immunizations, RAb16 nAb activity was directed against the 465 and 241/289 glycan holes. These specificities remained after the third BG505 SOSIP immunization but were broadened to include V1 after receiving the fourth BG505 SOSIP (Fig 3). nAb in RPc16 serum also targeted the 465 glycan hole at all time points but broadened to include the N611 epitope after the third and the 241/289 glycan hole after the fourth BG505 SOSIP immunization (Fig 3). Interestingly, the broadening of nAb targets between the third and fourth SOSIP immunizations was not associated with an increase in serum nAb titers against parental BG505 PV in either animal.

Three additional RM, 275_15, RHe16, and RUp16, had nAb ID_50_ titers less than 1:100 after the second BG505 SOSIP but greater than 1:100 after the third BG505 SOSIP. These time points were therefore tested against N611A only and the larger mutant PV panel, respectively. nAb against the N611A mutant PV was present in all three RM after the second BG505 SOSIP, indicating that this epitope was targeted early, while nAb against the parental BG505 Env was low to undetectable (Fig 3). After the third SOSIP immunization, all three RM had developed nAb targeting the 465 glycan hole that persisted after the fourth SOSIP. Targeting of N611 decreased to below the detection threshold in RHe16 and RUp16 with the subsequent boosts. Additionally, 275_15 transiently developed nAb against V1 after the third SOSIP and then shifted to the 241/289 glycan hole following the fourth SOSIP (Fig 3). Consistent with RAb16 and RPc16, nAb broadening in 275_15 was not associated with an increase in nAb titer against the parental BG505 PV. In contrast, nAb remained focused on the 465 glycan hole in the two RM that did experience an increase in ID_50_ titer between the third and fourth SOSIP, RHe16 and RUp16. This observation suggests that nAb against this target were boosted, which drove clonal expansion and affinity maturation in these RM. N611 antibodies also declined to below the threshold in RHe16 and RUp16 as nAb titers were increasing. Overall, antibodies with neutralization limited to the N611A mutant PV were detected in four of the five RM at one to two time points, and were present early in three RM, while neutralization of the parental Env PV was barely detectable. Subsequent SOSIP boosts in three RM resulted in shifting and broadening of nAb targets, which was not associated with an increase in potency against the parent PV. In contrast, focusing on a single epitope region, which was the 465 glycan hole in both RM analyzed, was associated with increasing nAb potency.

### Isolation of vaccine-induced potent autologous neutralizing monoclonal antibodies

**Fig 4:**
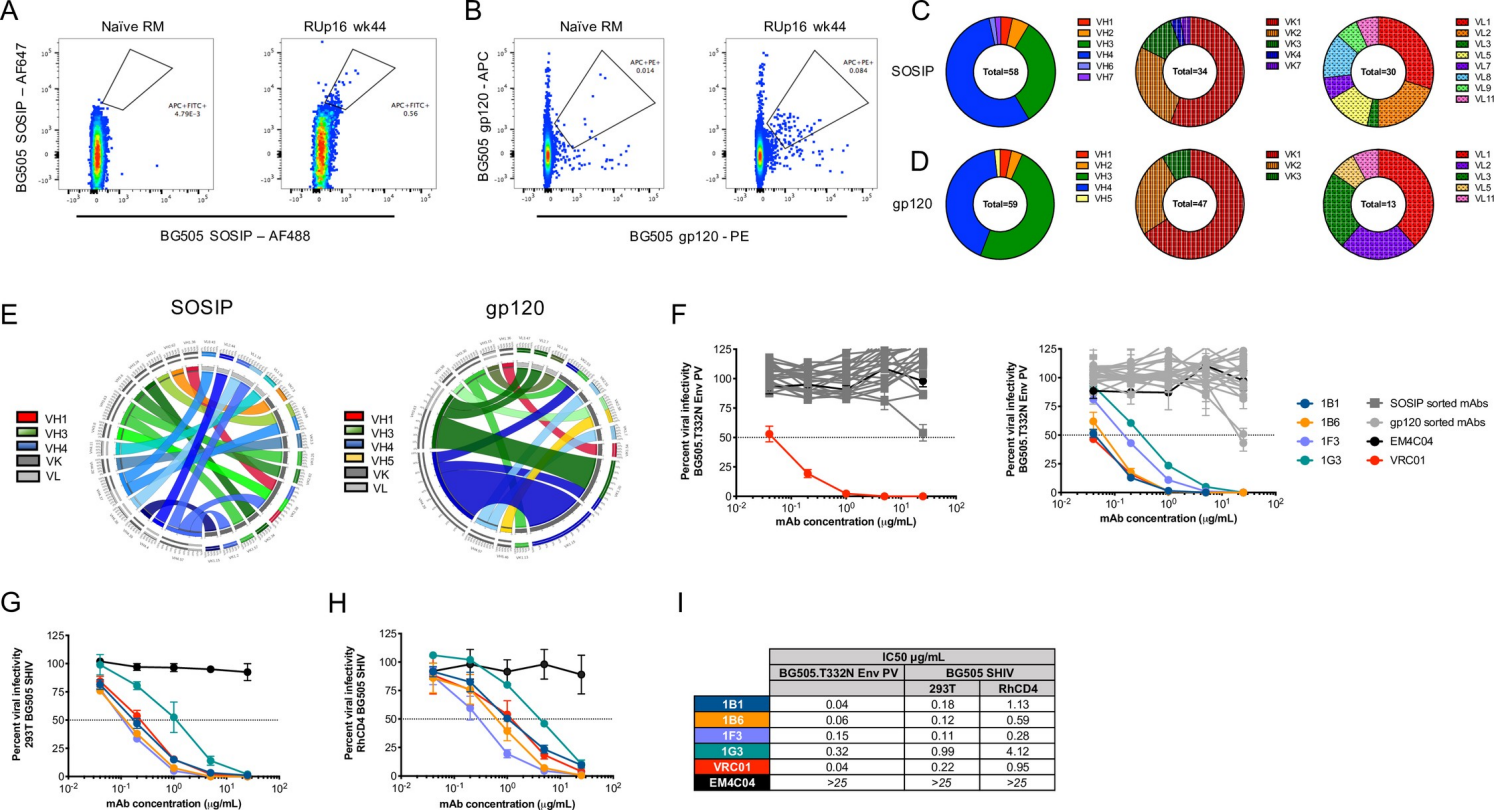
Isolation and characterization of mAbs derived from RUp16. Cryopreserved PBMC from RUp16 collected at week wk44 (four weeks after the third SOSIP immunization) were single-cell sorted as follows: size, live/dead, CD14^-^, CD3^-^, CD20^+^, IgG^+^, BG505 gp120^+^ or SOSIP^+^. Representative flow cytometry plots showing gates for (**A)** BG505 SOSIP-specific B cells and (**B)** BG505 gp120-specific B cells along with naïve RM controls, are shown. Variable regions were PCR amplified, sequenced, and germlines were assigned by analyzing the nucleotide sequences using IgBlast (https://www.ncbi.nlm.nih.gov/igblast/). The proportion of VH and VL kappa or lambda families are shown for (**C)** SOSIP-sorted and (**D)** gp120-sorted B cells with the gene families indicated by colors shown in the adjacent keys. CIRCOS diagrams were generated to illustrate VH and VK/L germline gene usage and pairing for (**E),** 18 SOSIP-sorted and 20 gp120-sorted mAbs from RUp16 that bound to BG505 SOSIP in ELISA. The VH and VL kappa or lambda germlines are shown on the left and right halves of the circle, respectively. The ribbons extending from VH to VL are color-coded based on VH family with kappa or lambda shown as shades of gray, as indicated in the key. The width of the band is proportional to the number of mAbs that were derived from the same VH and VL combination. (**F)** ELISA positive SOSIP-sorted (left) or gp120-sorted (right) mAbs were tested for neutralization of the BG505 Env PV in the TZM-bl assay. Infectivity is plotted against the concentration of mAb in μg/ml. The four neutralizing mAbs, as well as negative control anti-influenza HA mAb EM4C04 and positive control HIV bnAb VRC01, are shown in color as indicated in the key. Non-neutralizing mAbs are shown in shades of dark (SOSIP-sorted) or light (gp120-sorted) gray. The four neutralizing mAbs were also tested for neutralization of BG505.SHIV produced in (**G)** 293T cells and (**H)** RM CD4^+^ T cells in the TZM-bl assay. Each assay was run with duplicate wells and repeated independently at least twice. The error for each data point is indicated by the SEM. (**I)** The IC_50_ titers for each mAb against the BG505 Env PV and the BG505.SHIVs are shown in μg/ml. 25 μg/ml was the highest concentration tested.

For a higher resolution analysis of nAb associated with high titer and protection, we isolated mAbs from RUp16, a RM that was protected from ten challenges, developed the highest nAb titer, and persistently targeted the 465 glycan hole (Figs 1D, 1E, and 3). Notably, this RM also remained uninfected following a second set of six challenges initiated 20 weeks later, with a nAb ID_50_ titer of over 1:2000 [9]. Peripheral blood mononuclear cells (PBMC) cryopreserved at week 44 were sorted into single wells using fluorescently labeled BG505 SOSIP or gp120 to identify B cells specific to the antigen. The SOSIP and gp120-based sorts were performed independently so the genetic and functional characteristics of the recovered B cells and mAbs could be compared. We elected to include a sort with the gp120 probe to avoid isolating non-neutralizing antibodies directed against the trimer base [17]. A double labeling strategy was used for each probe to maximize discrimination between bona fide BG505 specific B cells and nonspecific background staining. Approximately 0.6% and 0.1% of the RUp16 IgG^+^ B cell population stained with BG505 SOSIP or gp120, respectively, compared to 0.01% or less of IgG^+^ B cells from a naïve RM (Fig 4A and 4B). Using the SOSIP probe, 58 Ig variable domain heavy chain (VH) sequences and 64 (34 kappa and 30 lambda) light chain (VL) sequences were recovered (Fig 4C). The gp120 probe produced 59 Ig VH and 60 VL (47 kappa and 13 lambda) sequences (Fig 4D). Interestingly, the SOSIP probe bound to B cells with more diverse VH and VL repertoires than the gp120 probe (Fig 4C and 4D), although these differences were not statistically significant (Chi Square test, p>0.05). Furthermore, the SOSIP probe bound to B cells with a significantly larger proportion of lambda light chains, than the gp120 sort (Fig 4C and 4D, Fisher’s exact test, p = 0.0045). However, consistent with previously published data, the antigen specific B cell response in this RM was dominated by VH3 and VH4 regardless of which probe was used [22,23].

Among all successfully sequenced VH and VL transcripts, we obtained 23 and 25 paired sequences from the BG505 SOSIP and gp120 probes, respectively. VH and VLs were cloned into expression plasmids and produced as 48 recombinant mAbs with a human IgG1 isotype constant region. Each mAb was tested in ELISA to confirm binding to BG505 SOSIP (S7 Fig). Of the 23 SOSIP-sorted antibodies generated, 78% bound to the BG505 SOSIP at levels above the negative control anti-influenza HA mAb EM4C04 when tested at 8 μg/ml. A similar proportion of gp120-sorted mAbs, 80%, bound to the BG505 SOSIP in ELISA. Thus, the recovery of trimer-specific mAbs and the magnitude of SOSIP binding were similar between the two sorting approaches (S7 Fig, p>0.05, Mann-Whitney). Circos diagrams of the VH and VL germline gene usage and pairing were generated for the 38 trimer binding mAbs recovered from both sorting approaches (Fig 4E). In the gp120-sorted mAbs, we noted two potential clonotypes, one derived from VH3.63/VK1.20 and another derived from VH4.39/VK1.15, using the NCBI IgBlast tool. Upon inspection of the VH and VL amino acid sequences, two of the three VH3.63-based sequences and four of the five VH4.39-based sequences were clonally related. In contrast, no clonotypes were observed in the SOSIP sorted mAbs (Fig 4E).

We next tested the ELISA-positive mAbs for neutralization of the parental BG505 Env PV. Four mAbs, obtained via the gp120 sort, exhibited potent autologous neutralization (Fig 4F). These four neutralizing mAbs, 1B1, 1B6, 1F3, and 1G3, comprised the VH4.39/VK1.15 clonotype shown in Fig 4E. The VH from the four mAbs were most likely derived from VH4.39, D2-2*01, and JH4*01, according to IgBlast, and each exhibited a nine amino acid CDRH3 (S8A Fig). The VLs were assigned the VK1.20 germline and JK3 region, with a 10 residue CDRL3 region. Due to the gaps in the RM germline database of IgBlast, we also queried the Rhesus macaque Germline Database (RhGLDB), which assigned IgHV4-AFB*01 to the VHs and LJI.RH_IGKV1.25 to the VLs. The VH sequences were between 9.4 and 12.8% mutated from VH4.39 and between 5.4 to 8.8% mutated from IgHV4-AFB*01. Likewise, the VK sequences were 7.5 to 8.5% mutated from VK1.20 and 4.0 to 6.7% mutated from LJI.RH_IGKV1.25. Inspection of the VH amino acid sequences aligned with each germline demonstrated that somatic hypermutation was focused in the CDRH1, CDRH2, and FRW3 regions (S8B Fig). In addition, each mAb had a unique CDRH3 sequence. Thus, regardless of the database used, the mAbs had acquired potent autologous neutralizing capacity through moderate somatic hypermutation in VH and VL.

**Fig 5:**
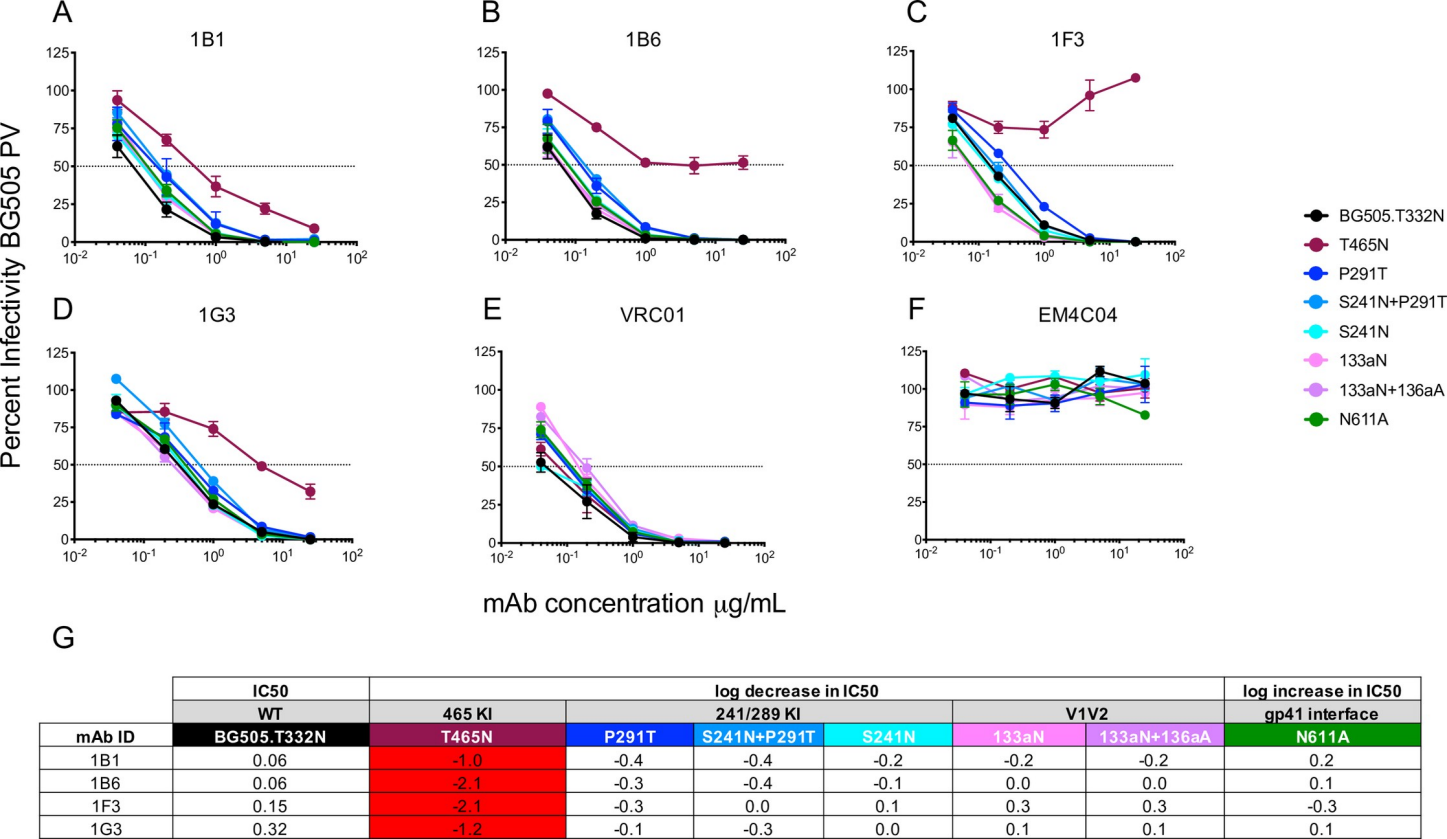
Mapping of mAb neutralizing activity. Neutralizing activity against the parental BG505 and mutant PVs was evaluated using the TZM-bl assay in (**A—F)**. The mAb is indicated above each graph, which displays percent infectivity plotted against mAb concentration in μg/ml. mAbs VRC01 (**E)** and EM4C04 (**F)** were used as positive and negative controls, respectively. (**G)** The log10 fold increase or decrease in IC_50_ titer of each mutant PV relative to the parental BG505 PV is shown. An increase in IC_50_ of greater than 0.5log10 is highlighted red.

mAbs 1B1 and 1B6 displayed exquisite potency against the BG505 Env PV, with nAb IC_50_ titers of 0.04 and 0.06 μg/ml, which are comparable to VRC01 (IC_50_ = 0.04μg/ml) (Fig 4F and 4I). The remaining clonal variants, 1F3 and G3, were less potent, with IC_50_ titers of 0.15 and 0.32 μg/ml (Fig 4F and 4I). These mAbs also potently neutralized the challenge virus BG505.SHIV, whether produced in 293T cells or in RM CD4^+^ T cells, the latter of which was the stock used in the efficacy trial (Fig 4G–4I). The number of mutations from germline did not correlate with mAb neutralization potency, indicating that the nature and position of the mutations is important. We next used mutant PVs from the panel to probe the mAb specificities. All four mAbs targeted the 465 glycan hole (Fig 5), as did the serum nAb for this RM at the corresponding time point (Fig 3) and at the time of challenge (Fig 1D). Interestingly, each mAb exhibited a different capacity to neutralize the T465N mutant. 1B1 and 1G3 neutralized the T465N mutant PV less efficiently than the BG505 parent but with relative potency (Fig 5A and 5D). However, 1B6 reached a distinct plateau, unable to neutralize more than 50% of viral infectivity above 1 μg/ml (Fig 5B). Finally, 1F3 had no neutralizing activity against the T465N mutant PV even at 25 μg/ml (Fig 5C). Even though RUp16 serum did not exhibit heterologous neutralization breadth when tested against a small panel of heterologous Env PVs, we tested the most potent mAbs 1B1 and 1B6 for neutralizing activity against five HIV-1 Env PVs that exhibited varying patterns of N-linked glycosylation adjacent to residue 465, including some that were similar to BG505 (S9 Fig). Nevertheless, neither mAb showed any heterologous neutralization breadth. These observations suggest that the presence or absence of glycans adjacent to residue 465 is not the only determinant of neutralization, and is consistent with both the heterogeneity within this epitope region and among the antibodies directed towards it. The partial resistance of T465N to neutralization by RUp16 serum (Fig 1D) also reflects the varying patterns of T465N neutralization among the mAbs. Isolation and analysis of these four potently neutralizing, clonally related mAbs after the third SOSIP immunization suggest that serum nAb in this RM was mediated by an expanded clonotype directed at 465 glycan hole-proximal epitopes. It will be important to define the sites of contact for these mAbs on BG505 SOSIP to understand how this lineage arose and expanded, as it is likely that these mAbs are representative of those that contributed to the prevention of SHIV infection through two challenge series.

## Discussion

To date, HIV vaccines have not elicited antibodies in humans that are able to neutralize autologous or heterologous variants [24]. Recent pre-clinical efforts have been devoted to developing vaccine regimens that elicit durable, high-titer autologous nAb in nonhuman primates using native-like trimers [3,5]. Our previous study also suggested that cellular immunity against SIV Gag can contribute to protection when high titer nAb activity is absent [9]. Here we focused on RM with high serum nAb ID_50_ titers on day of challenge that were unequivocally associated with protection across groups. We demonstrate that nAb vigorously targeted the C3/465 glycan hole cluster, with varying effects of mutations in this region in protected RM. Furthermore, we show that in a protected RM with an exceptionally high serum nAb ID_50_ titer, a potently neutralizing antibody clonotype that also targeted this region arose within the heterogeneous population of BG505 SOSIP elicited antibodies, which included neutralizing and non-neutralizing specificities [12,16,17,20].

nAb epitope mapping at the serum level revealed that one or more sites within the C3/465 glycan hole cluster was involved in serum neutralization in all protected, high titer RM. This is consistent with other studies that have mapped nAb responses in RM, although more frequent targeting of the 241/289 glycan hole and V1 was also observed here [6,10,14,15]. These discrepancies could reflect differences in the immunogen, threshold, particulars of the assay and PVs, and genuine heterogeneity among immunized RM. We found that protected, high titer RM targeted between one and three major neutralization epitopes using a 0.5log10 reduction in ID_50_. In all cases, the mutants were not completely resistant to neutralization. The partial neutralization of mutants could be due factors such as the polyclonal nature of serum nAb, incomplete coverage of the surface by the introduced glycans, sequon skipping, or allosteric changes the BG505 Env from addition of a glycan [25–27]. No advantage to targeting a certain combination of epitopes, or earlier vs later in the vaccination regimen, was noted (Figs 1 and 2). However, focusing on a single region during the vaccination regimen was associated with an increase in nAb titer, whereas broadening or shifting of nAb targets was not. In two RM where increasing titers were observed between the third and fourth SOSIP immunizations, the target was the 465 glycan hole. Antibodies against the N611 region in high titer, protected RM were present while serum nAb was still developing. Interestingly, these non-neutralizing antibodies were more prevalent in RM with low nAb titers on the day of challenge, whether protected or infected. Thus, persistently targeting this region in gp41 that is shielded in the parental BG505 Env could potentially impact nAb titers and protection, although this finding requires further investigation.

We also interrogated antibody binding to the BG505 SOSIP by nsEMPEM to determine whether binding to a specific region or regions on the BG505 SOSIP was associated with protection or nAb titer. However, no pattern was revealed. Antibody specificities detected by nsEMPEM generally overlapped with the serum nAb mapping; however, in a few instances, nAb specificities did not appear in the nsEMPEM analysis, and vice versa. While this observation is possibly due to the inherent difficulties in both techniques, it also reiterates that SOSIP binding is not always instructive for neutralization, as there are some inevitable differences between native and near native Env conformations, including base exposure, sequon skipping, and other structural differences [25–27]. Furthermore, given the pervasive presence of co-circulating antibodies binding to four to six specificities, it is likely that their presence impacts recognition of nAb epitopes on the immunogen during repeated boosts *in vivo*. The variation in antibody targeting highlights the stochastic nature of B cell responses in an immunized outbred population and suggests that re-directing responses away from the immunogenic trimer base and glycan holes towards more viable nAb epitopes (i.e. CD4 binding site) is challenging.

Overall, we show that RM that developed high titers of nAb vigorously targeted the C3/465 glycan hole cluster, and these specificities likely contributed to protection. However, glycan hole epitopes are generally unique to BG505, and thus autologous nAb has no mechanism by which to acquire breadth. The development of potent autologous nAbs is, however, an important first step in the generation of antibodies that could be driven to acquire heterologous neutralization breadth through targeted immunogens. With a more complete understanding of the 465 epitope region and other immunogenic epitopes, it is possible that strategically glycosylated Env variants could be used to guide nAb to acquire neutralization breadth. It could also be that other Envs with more complete glycan coverage may be better suited as stabilized trimer immunogens [28–30]. The expansion of a clonal family with exceptional potency that is on par with VRC01 demonstrates that nAb against a single epitope region can be driven to expand and mature by homologous boosting, and that powerful adjuvants [31,32] such as 3M-052 could also be an important vaccine component. Remarkably, these vaccine elicited neutralizing mAbs had moderate levels of somatic hypermutation in VH up to 8–12% from germline, depending on the database used. Four neutralizing mAbs from this family were recovered out of 25 gp120-sorted mAbs total, suggesting that this lineage was present at a relatively high frequency following the third SOSIP immunization, and likely are representative of the nAbs that contributed to protection in this RM. Notably, these mAbs also neutralized the SHIV challenge virus, and the serum nAb activity that developed in this RM was exceptionally durable [9]. Understanding how this lineage arose, identifying and characterizing the unmutated common ancestor and intermediates, and determining whether other members of the clonotype went ‘off track’, could provide clues for eliciting a more consistent potent and durable nAb response. In addition, it will be important to determine whether similar clonotypes arose in other high titer, protected RM whose serum nAb strongly targeted the same epitope region. Our study provides new evidence that multiple SOSIP immunizations can expand potent strain-specific nAb lineages against a single target region, a desirable feature for vaccines that can be potentially further developed by strategies that selectively fill in immunogenic glycan holes with subsequent boosts, incorporate powerful adjuvants, and test Envs that have more complete glycan coverage as immunogens.

## Materials and methods

### Ethics statement

The RM immunization experiment from which the serum samples were derived have been described previously [9]. The study was approved by the Institutional Animal Care and Use Committee (IACUC) at Emory University and was in compliance with NIH guidelines. Animal research was also in compliance with the Animal Welfare Act and other Federal statutes and regulations relating to experiments involving animals. All animal research adhered to the principles stated in the 2011 Guide for the Care and Use of Laboratory Animals prepared by the National Research Council. Yerkes National Primate Research Center (YNPRC) is fully accredited by the Association for Assessment and Accreditation of Laboratory Animal Care (AAALAC). Methods of euthanasia were consistent with the American Veterinary Medical Association with Guidelines.

### RM samples

Blood samples were obtained from a total of 30 Indian rhesus macaques (*Macaca mulatta*) immunized during a vaccine efficacy study carried out previously at the Yerkes National Primate Research Center [9]. The samples analyzed here included serum and PBMC. The study utilized female RM that were 3–15 years of age and confirmed negative for SIV infection. The immunization regimen has been previously described [9]. A description of the RM serum included in each assay is provided in S2 Table.

### Measurement of antigen specific serum IgG

Antigen specific IgG was measured using an enzyme-linked immunosorbent assay (ELISA) using BG505 SOSIP.664.T332N gp140 protein, captured on Nunc MaxiSorp microtiter plates (439454, Thermo Fisher Scientific) as previously described with modifications [33]. Briefly, plates were coated with the BG505 SOSIP.664.N332 gp140 protein at 2 μg/ml in PBS and incubated overnight at 4°C. The following day, plates were washed, blocked for 1 h with blocking buffer (5% milk powder (170–6404, BioRad) dissolved in 4% whey (W1500, Sigma) buffer prepared in PBS containing 0.05% tween-20 (PBST) and incubated for 2 h with 3-fold dilutions of serum. For the standard, known concentrations of purified serially diluted RM IgG (PR-2406, Nonhuman Primate (NHP) Reagent Resource) were captured using goat anti-RM IgG (617-101-012, Rockland Immunochemicals). Bound IgG was detected using peroxidase-conjugated anti-RM IgG (GAMon/IgG(Fc)/PO, Nordic-MUbio, Susteren, The Netherlands) and tetramethylbenzidine substrate (5120–0047, KPL). The reaction was stopped by adding 100 μl of 1N H_3_PO_4_. Each plate included a standard curve and absorbance was measured at 405nm. Standard curves were fitted and sample concentrations interpolated as micrograms of antibody per milliliter of serum using SOFTmax 2.3 software (Molecular Devices).

### ELISA screening of mAbs

Binding of mAbs to the BG505 SOSIP.664.T332N gp140 protein was confirmed by ELISA as previously described with modifications [6]. Nunc MaxiSorp 96-well flat bottom plates (44-2404-21, Thermo Fisher Scientific) were coated overnight with 2.5 μg/ml streptavidin (434302, Invitrogen) in PBS at 50 μl per well. Plates were then washed three times in PBST and blocked with PBS + 3% BSA for 1 h at room temperature. To capture biotinylated BG505 SOSIP.664.T332N-Avi gp140, protein was diluted to a concentration of 1 μg/ml in PBS + 3% BSA and immobilized on streptavidin-coated 96-well plates for 2 h at RT. Plates were then washed three times with PBST and serially diluted mAbs in PBS + 3% BSA were then added for 1 h at RT. Plates were then washed three times in PBST and HRP conjugated goat anti-human IgG (109-035-088, Jackson ImmunoReseach Laboratories) was added for 1 h at 1:10,000 in PBS + 3% BSA at RT. Bound IgG was detected using the tetramethylbenzidine substrate (KPL, 52-00-03) and the reaction was stopped by adding 100 μl of 2M H_2_SO_4_ (4N, SA818-1, Thermo Fisher Scientific). All plate washes were done using a plate washer with stacker and absorbance was measured at 405nm using an EPOCH microplate reader (BioTek).

### Measurement of fusion peptide specific IgG

Fusion peptide (FP) ELISA assays were performed exactly as for the ELISA screening of mAbs, with the following modifications. FP specific IgG was measured using NiNTA plates (35061, Qiagen). NiNTA plates were blocked with PBS + 5% BSA for 2 h at RT. After blocking, the C-terminally his-tagged BG505 FP peptide, residues 512–520 based on HXB2, (AVGIGAVFLG-HHHHHH) was added at 2.5 μg/ml in PBS + 5% BSA and incubated for 2 h at RT. RM serum samples were serially diluted in PBS + 5% BSA, added to the FP coated wells, and incubated for 2 h at RT.

### TZM-bl Neutralization assays

Neutralization against HIV-1 Env BG505.T332N (derived from BG505.W6M.Env.C2, Genbank accession #DQ208458), BG505.W6M.C2 containing T332, and ten HIV-1 BG505.T332N mutants described in [5,12,19] were measured using serially diluted, heat-inactivated immunized RM serum or mAbs in the TZM-bl assay as previously described, using cells plated one day prior to the assay [28,32,34–46]. In brief, Env PV was generated by transfecting the Env-expressing plasmid DNA alongside the HIV-1 SG3ΔEnv proviral backbone DNA into 293T cells, using the Fugene HD reagent as recommended (E2312, Promega). PV stocks were collected from the 293T cell supernatants at 48–72 h after transfection, clarified by centrifugation, divided into small volumes, and frozen at –80°C. 2000 IU of each titered Env PV (in DMEM with ∼3.5% (vol/vol) FBS (SH30070.03, Hyclone) and 40 μg/ml DEAE-dextran) was mixed with five-fold serial dilutions of heat-inactivated serum samples and assayed for inhibition of the Env PV using the TZM-bl indicator cell line, with luciferase as the readout. At 48 h post-infection, the cells were lysed and luciferase activity was measured using a Cytation3 multimode microplate reader (BioTek). The average background luminescence from a series of uninfected wells was subtracted from each experimental well. Experimental wells were compared against virus without a test reagent (100% infectivity). All assays contained duplicate wells and were repeated at least once independently. Neutralization ID_50_ or IC_50_ titer values were calculated in Graphpad Prism using the dose–response inhibition analysis function with variable slope, log-transformed x values, and normalized y values.

### Fold change in neutralization of mutant compared to parent BG505 PV

The raw ID50 values used to calculate the fold change in neutralization are shown in S1 Table. For fold change of the mutant PVs compared to the parental BG505 PV, the following calculations were applied. To calculate a fold reduction in serum ID_50_ titer, the parent ID_50_ was divided by the glycan addition mutant ID_50_, and the log10 of this number was reported. To calculate a fold increase in serum ID_50_ titer, the mutant ID_50_ (T357A, N398A, and N611A) was divided by the parent ID_50_, and the log10 of that ratio was reported. For fold increase in mAb IC_50_ titer, the glycan addition mutant IC_50_ was divided by the parent IC_50_ and the log10 of the number was reported. For the fold decrease in IC_50_ titer, the parent IC_50_ was divided by the mutant IC_50_ (133aN, 133aN + 136aA, P291T, P291T + S241N, S241N, T465N, and TI357KT) and the log10 of this ratio was reported. A fold change of 05.log10 or greater was considered to be substantial resistance to or enhancement of neutralization susceptibility.

### Negative stain electron microscopy polyclonal epitope mapping

Serum IgG from eight RM was purified using a protein G spin plate (45204, Thermo Fisher Scientific). Purified polyclonal serum IgGs were digested into Fabs using immobilized papain resin (20341, Thermo Fisher Scientific) as previously described (14). Undigested IgG and Fc were removed using Protein A resin (17-1279-03, GE Healthcare). 1 mg of polyclonal Fab was complexed with 15 μg BG505 SOSIP.664.T332N gp140 protein overnight at room temperature. Unbound Fabs were removed using size exclusion chromatography on a Superose 6 Increase 10/300 column (29-0915-96, GE Healthcare). Purified BG505 SOSIP.664/polyclonal Fab complexes were diluted to 0.03 mg/ml in TBS and applied to freshly glow discharged carbon-coated 400-mesh copper grids (EMS400-CU, Electron Microscopy Sciences) and blotted after 10 seconds. The grids were then stained with 3 μl of 2% (w/v) uranyl formate, immediately blotted, and stained again for 30–45 seconds followed by a final blot. Image collection and data processing was performed as described previously on a FEI Talos microscope operating at 200 keV and equipped with a Ceta 16M camera (1.98 Å/pixel; 72,000× magnification) with an electron dose of ∼25 electrons/Å^2^ using Leginon [47,48]. 2D classification, 3D sorting and 3D refinement conducted using Relion v3.0 [49]. EM density maps were visualized using UCSF Chimera and segmented using Segger [50,51].

### Antigen-specific B cell sorting

BG505 gp120 with a 6xHis tag (gp120 BG505.W6M.ENV.C1; IT-001-176, Immune Technology Corporation) and biotinylated, Avi-tagged BG505 SOSIP.664.T332N gp140 protein were used for single B cell sorting. Biotinylated BG505 SOSIP was conjugated in a 2:1 ratio to streptavidin-conjugated AF647 (405237, BioLegend) and AF488 (405235, BioLegend), resulting in a fluorescently labeled probe. Week 44 cryopreserved PBMC from RUp16 (∼10 million cells per sort) were washed and resuspended in PBS + 2% FBS. After counting, cells were incubated with BG505 gp120-His or biotinylated SOSIP and stained with: live/dead fixable viability dye eFluor 780 (65-0865-14, eBioscience,), anti-CD14 PE-Cy7 (367111, BioLegend), anti-CD3 Pacific Blue (317313, BioLegend), anti-CD20 BV650 (302335, BioLegend), anti-IgG FITC (555786, BD Pharmingen) or anti-IgG PE (4700–0, Southern Biotech). Anti-His PE (130-120-718, Miltenyi Biotec) and anti-His APC (130-119-782, Miltenyi Biotec) were used to detect the gp120 probe. Sorts were carried out using a FACS Aria Cell Sorter and the following gating strategy: size, singlets, live, CD14^-^, CD3^-^, CD20^+^, IgG^+^. Live CD20^+^ IgG^+^ B cells that were double positive for BG505 gp120 (PE and APC) or SOSIP (AF488 and AF647) were single cell sorted into 96-well plates containing 20 μl cell lysis buffer (Superscript III RT buffer, Tween, DTT, 18080–044, Invitrogen) and RNaseOUT (1077019, Invitrogen). Plates were temporarily placed on dry ice before moving to -80°C for storage. Full-length cDNA amplification of single cells was performed using a modified version of the SMART-Seq II protocol [52], as previously described [53]. The analysis of the surface markers of the positive cells was performed on FlowJo.

### Antibody cloning

Immunoglobulin heavy and light chain variable domain regions were PCR-amplified as previously described, using oligo-dT (AM5730G, Thermo Fisher Scientific) and SuperScript III (18080–044, Invitrogen) [22,54]. Five μl of the RT reaction was used to amplify heavy (IgG only), kappa, and lambda chain variable regions using high performance liquid chromatography (HPLC) purified primers as described in Sundling et al. [22] and Phusion Hotstart II High Fidelity DNA Polymerase (F537S, Thermo Fisher Scientific) for first round PCR. For the second round PCR reaction, 2.5 μl of the first round was used as a template in addition to external primers used in Liao *et al*. [54]. PCR amplified variable regions were gel purified (28706, Qiagen) and combined with a CMV promoter containing DNA fragment, and the appropriate corresponding human constant region DNA fragment (including a polyA tail) via overlapping PCR [54]. Plasmids containing the CMV promoter (HV0024), the heavy chain IgG1 constant region (HV0023), the kappa Ig constant region (HV0025), and the lambda Ig constant region (HV0026) were provided by the Duke Human Vaccine Institute. The assembled full-length heavy and light chain segments were then cloned into pCR2.1TOPO-TA (K4500-40, Thermo Fisher Scientific) for long-term storage and mAb expression. VH and VL plasmid pairs were co-transfected at a 1:2 ratio into 6 well plates containing Expi293F cells (A14635, Thermo Fisher Scientific). Seven days post-transfection, mAbs were purified from the cell culture supernatant using Ab SpinTrap with Protein G Sepharose High Performance (2840834, GE Healthcare). The concentrations of the purified mAbs were quantified on an Octet RED96 using Anti-Human IgG Fc AHQ biosensors (18–5001, ForteBio) as described [38].

### Assignment of Ig germlines

Germlines for heavy, kappa, and lambda sequences were initially assigned using the query tool on NCBI IgBlast (https://www.ncbi.nlm.nih.gov/igblast/). Nucleotide sequences were submitted and the germline with the highest identity was used to assign putative germlines, which were then used to determine the VH and VL kappa or lambda family frequencies and heavy and light chain pairing. For the four neutralizing mAbs, the nucleotide sequences were also submitted to the RhGLDB (http://ward.scripps.edu/gld/)). The amino acid sequences were used to define the framework and complementarity determining regions of the heavy and light chain sequences using IMGT V-QUEST (http://www.imgt.org/IMGT_vquest/input). Geneious v9.0.4 and SeqPublish (https://www.hiv.lanl.gov/content/sequence/SeqPublish/seqpublish.html) were used to calculate pairwise identity and generate alignments.

## Supporting information

S1 Fig Assessment of serum binding and autologous neutralization titers in RM. **(A)** The immunization schedule for the UM1 vaccine efficacy study is plotted along a timeline in weeks, with key time points indicated. The vaccination arms of the trial are shown on the left and the agents used for immunization are indicated at the top. SOSIP indicates BG505 SOSIP.664.N332. The adjuvant used was 3M-052. HVV = heterologous viral vectors, each expressing SIVmac239 Gag (no Env); VSV-Gag = vesicular stomatitis virus; VV-Gag = vaccinia virus; Ad5-Gag = adenovirus type 5. Ten low dose repeated intra-vaginal challenges using SHIV.BG505 were carried out weekly beginning at week 84. BG505 SOSIP-specific serum IgG measured by ELISA on the day of challenge (week 84) and is shown for (**B)** vaccine groups (p>0.05) and (**C)** protected vs. infected RM (p>0.05). There was no significant difference between vaccine or challenge outcome groups using the Mann-Whitney test. (**D)** Neutralization activity against BG505 Env PV is shown for the vaccination groups, and was not significantly different using a Mann-Whitney test (p>0.05). A significant correlation was observed between serum nAb titers measured at week 84 against BG505 Env PV T332N and the T332 version (Spearman’s Rank, r = 0.7932, p<0.0001). ID_50_ titers were calculated using GraphPad Prism. For (**B)** through (**D)**, the horizontal bar represents the median for each group.
(TIF)

S2 Fig BG505 Env PV mutants used to map serum neutralization targets. **(A)** Env PVs containing mutations that affect four major nAb targets on the BG505 Env were used (HXB2 numbering): V1 residues 133/136, a glycan hole located near positions 241 and 289, the C3/465 glycan hole cluster, and a region proximal to N611 near the trimer base. The substitutions that shift or add a glycan motif in BG505 Env generally result in a reduction in neutralization sensitivity; removal of the N355 (T357A), N398, and N611 glycan motifs from the BG505 Env generally results in an increase in neutralization sensitivity. (**B)** Amino acid residues at positions 131–137 in V1 of the BG505 WT and the 133aN and 133aN + 136aA mutants shows the shift of a glycan and elongation of the V1 loop. (**C)** Amino acid residues 355–358 in C3 for BG505 WT and the TI357KT mutant show a glycan shift and sequence changes. Glycan motifs are highlighted in gray, with bolded text, and mutations or insertions are indicated in red.
(TIF)

S3 Fig Mapping of bnAb neutralizing activity. HIV broadly neutralizing antibodies were used to compare the sensitivity of the BG505.T465N mutant to BG505 Env PV in the TZM-bl assay. A heat map of the IC_50_ titers for each bnAb are shown in μg/ml, with 25 μg/ml being the highest concentration tested. The negative control anti-influenza HA mAb EM4C04 was included. Red to yellow indicates high to moderate susceptibility; yellow to green indicates resistance.
(TIF)

S4 Fig Sequence alignment of major HIV-1 clades and CRFs proximal to residue 465 in Env. The amino acid sequence alignment contains BG505.SOSIP.664.gp140 (immunogen), BG505.W6M.Env.C (the parental PV), the BG505.T465N mutant, and a consensus for HIV-1 group M, all major HIV-1 clades and CRFs, with HXB2 as a reference sequence (Genbank K03455). The alignment was generated using https://www.hiv.lanl.gov/content/sequence/NEWALIGN/align.html and https://www.hiv.lanl.gov/content/sequence/SeqPublish/seqpublish.html. N-linked glycan motifs within this region are highlighted in gray and the T465N mutation is indicated in with red text. Dashes indicate conserved residues, while differences are shown, except within the 465 adjacent region, where glycan motifs are indicated by showing the amino acid residues (NXS/T where X is any residue except proline). Dots indicate a gap.
(TIF)

S5 Fig Comparison of non-neutralizing antibodies against N611 epitopes on day of challenge. Serum neutralizing activity on the day of challenge (week 84) was evaluated against the parental BG505 and N611A mutant PVs using the TZM-bl assay for all but one immunized RM (n = 29). The log10 increase in ID_50_ titer of the N611A mutant compared to the parental BG505 Env is shown in vertical plots for each RM grouped by vaccination, challenge outcome, and titer/challenge outcome (**A—C**). Red symbols indicate HVV + SOSIP immunized RM; black symbols indicate SOSIP immunized RM. (**A)** vaccination groups HVV + SOSIP vs SOSIP (p>0.05), (**B)** protected vs infected (p = 0.047), and (**C)** high titer protected vs. low titer protected (p = 0.006) and low titer infected (p = 0.004) are shown. Mann-Whitney tests were used to perform the two group comparisons in (**A)** and (**B)** and a Kruskal-Wallis test with Dunn’s correction was used for the three-group comparison in (**C)**. All were performed using GraphPad Prism (*p<0.05, **p<0.01). Horizontal bars in (**A—C)** represent the median of the group. The dashed lines indicate the threshold of 0.5log10 fold increase over the parental BG505.
(TIF)

S6 Fig Assessment of serum binding to BG505 fusion peptide. BG505 fusion peptide specific IgG was measured by ELISA on the day of challenge (week 84) using serum from 30 immunized RM. His-tagged fusion peptide was captured onto the plate wells. Each ELISA was run with duplicate wells and repeated independently at least twice. The horizontal bars indicates the median. Filled symbols indicate the positive control, bnAb VRC34 (red), and negative control anti-influenza HA mAb, EM4C04 (black).
(TIF)

S7 Fig Binding of SOSIP and gp120 sorted mAbs to BG505 SOSIP. An ELISA was performed using 18 SOSIP-sorted and 20 gp120-sorted mAbs to measure binding to BG505 SOSIP, which was captured onto the plate wells. The absorbance was measured using each mAb at 8 μg/ml. Each ELISA was run with duplicate wells and repeated independently at least twice. Horizontal bars indicate the median. Filled black symbols indicate the negative control anti-influenza HA mAb, EM4C04.
(TIF)

S8 Fig Amino acid sequence of RUp16 neutralizing mAbs. The nucleotide sequences of the variable domains of the four neutralizing mAbs were submitted to IgBlast, the VDJ and VJ assignments for the heavy and light chain, respectively, are shown in (**A**) The nucleotide identity to the assigned germline from IgBlast, and from a second database RhGLDB in parenthesis, is also shown for each mAb. Amino acid alignments of the four heavy chains with putative germlines, IGHV4-AFB*01 (RhGLDB) and VH4.39 (IgBLAST) in (**B)** and the four light chains with putative germlines, LJI.RH_IGKV1.25 (RhGLDB) and VK1.20 (IgBLAST) in (**C)** are shown. Identity with the germline is indicated by a dash, and amino acid differences are shown. The framework (FRW) and complementarity determining regions (CDR, shaded) are indicated above the sequences. Dots indicate that the sequence was not present in the germline (CDRH3 and CDRL3).
(TIF)

S9 Fig Assessment of heterologous neutralization of RUp16. The sequence alignment of heterologous HIV-1 Envs proximal to residue 465 are shown in **(A)**. The amino acid sequence alignment contains BG505.W6M.Env.C (DQ208458, clade A), and HIV-1 Envs CH119 (EF117271, CRF07), TRO11 (AY835445, clade B), 246F3 (HM215279, clade AC), BJOX2000 (HM215364), CRF07), CE0217 (FJ443575, clade C), CE1176 (FJ444437, clade C), with HXB2 (K03455) shown as a reference sequence. The alignment was generated using Geneious v9. N-linked glycan motifs within this region are highlighted in gray. Dashes indicate conserved residues, while differences are shown, except within the 465 adjacent region, where glycan motifs are indicated by showing the amino acid residues (NXS/T where X is any residue except proline). Dots indicate a gap in the alignment. The TZM-bl assay was used to assess neutralizing activity against the panel of PVs using serum **(B)** and mAbs **(C)** from RUp16 following the third protein immunization. **(B)** A key indicating the Envs used to evaluate serum neutralizing activity is shown to right of the graph. The reciprocal of the serum dilution is plotted on the x axis on a log10 scale and the percent of viral infectivity is plotted on the y axis relative to the virus only control at 100%. **(C)** Neutralizing mAbs isolated from RUp16 were tested against the Envs indicated above each graph. The percent of viral infectivity is plotted against mAb concentration in μg/ml. For comparison, murine leukemia virus (MLV) Env was included as a negative control **(B and C)**. mAbs VRC01 and EM4C04 were used as positive and negative controls, respectively.
(TIF)

S1 Table ID_50_ titers of BG505 mutants compared to the parental Env. (TIF)

S2 Table Overview of RM vaccine groups, challenge status, ID_50_ titers, and mapping analyses. (TIF)
