# Supplementary figures and images for: The C3/465 glycan hole cluster in BG505 HIV-1 envelope is the major neutralizing target involved in preventing mucosal SHIV infection
Source: PLoS Pathog. 2021 Feb 8;17(2):e1009257. doi: 10.1371/journal.ppat.1009257 (PMC7895394; doi:10.1371/journal.ppat.1009257)

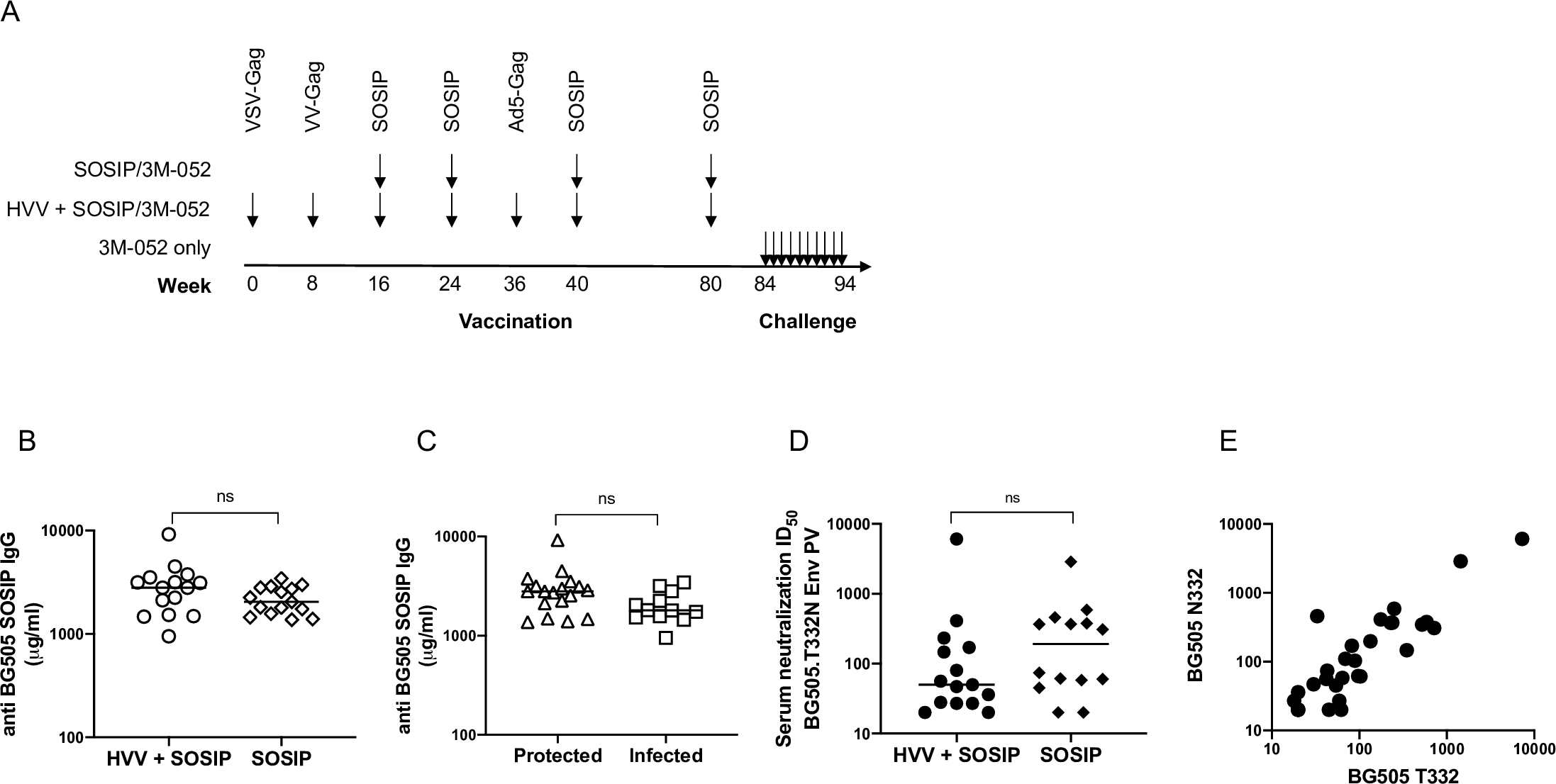

Supplement: S1 Fig — (A) The immunization schedule for the UM1 vaccine efficacy study is plotted along a timeline in weeks, with key time points indicated. The vaccination arms of the trial are shown on the left and the agents used for immunization are indicated at the top. SOSIP indicates BG505 SOSIP.664.N332. The adjuvant used was 3M-052. HVV = heterologous viral vectors, each expressing SIVmac239 Gag (no Env); VSV-Gag = vesicular stomatitis virus; VV-Gag = vaccinia virus; Ad5-Gag = adenovirus type 5. Ten low dose repeated intra-vaginal challenges using SHIV.BG505 were carried out weekly beginning at week 84. BG505 SOSIP-specific serum IgG measured by ELISA on the day of challenge (week 84) and is shown for (B) vaccine groups (p>0.05) and (C) protected vs. infected RM (p>0.05). There was no significant difference between vaccine or challenge outcome groups using the Mann-Whitney test. (D) Neutralization activity against BG505 Env PV is shown for the vaccination groups, and was not significantly different using a Mann-Whitney test (p>0.05). A significant correlation was observed between serum nAb titers measured at week 84 against BG505 Env PV T332N and the T332 version (Spearman’s Rank, r = 0.7932, p<0.0001). ID50 titers were calculated using GraphPad Prism. For (B) through (D), the horizontal bar represents the median for each group. (TIF) [file ppat.1009257.s001.tif]

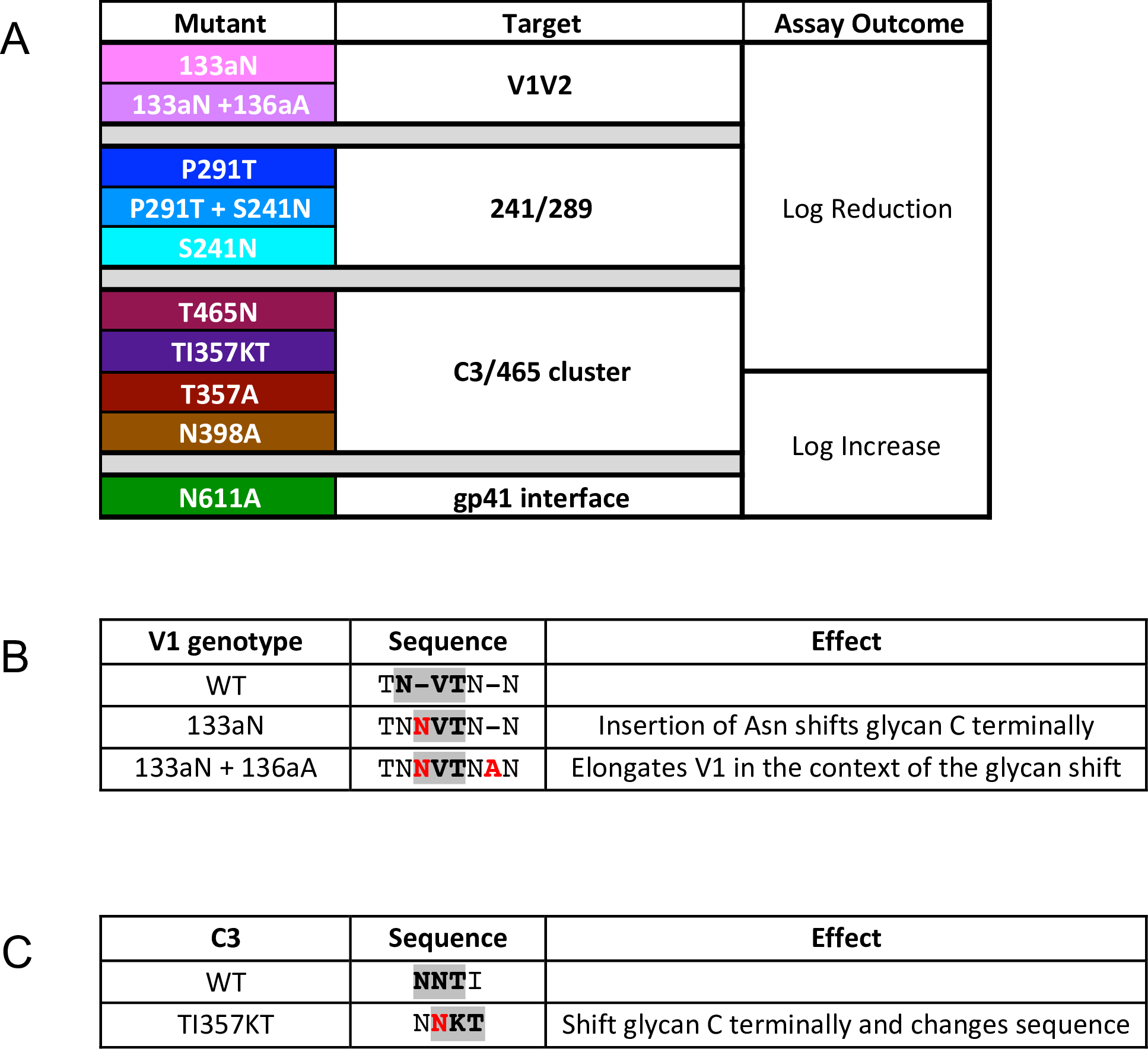

Supplement: S2 Fig — (A) Env PVs containing mutations that affect four major nAb targets on the BG505 Env were used (HXB2 numbering): V1 residues 133/136, a glycan hole located near positions 241 and 289, the C3/465 glycan hole cluster, and a region proximal to N611 near the trimer base. The substitutions that shift or add a glycan motif in BG505 Env generally result in a reduction in neutralization sensitivity; removal of the N355 (T357A), N398, and N611 glycan motifs from the BG505 Env generally results in an increase in neutralization sensitivity. (B) Amino acid residues at positions 131–137 in V1 of the BG505 WT and the 133aN and 133aN + 136aA mutants shows the shift of a glycan and elongation of the V1 loop. (C) Amino acid residues 355–358 in C3 for BG505 WT and the TI357KT mutant show a glycan shift and sequence changes. Glycan motifs are highlighted in gray, with bolded text, and mutations or insertions are indicated in red. (TIF) [file ppat.1009257.s002.tif]

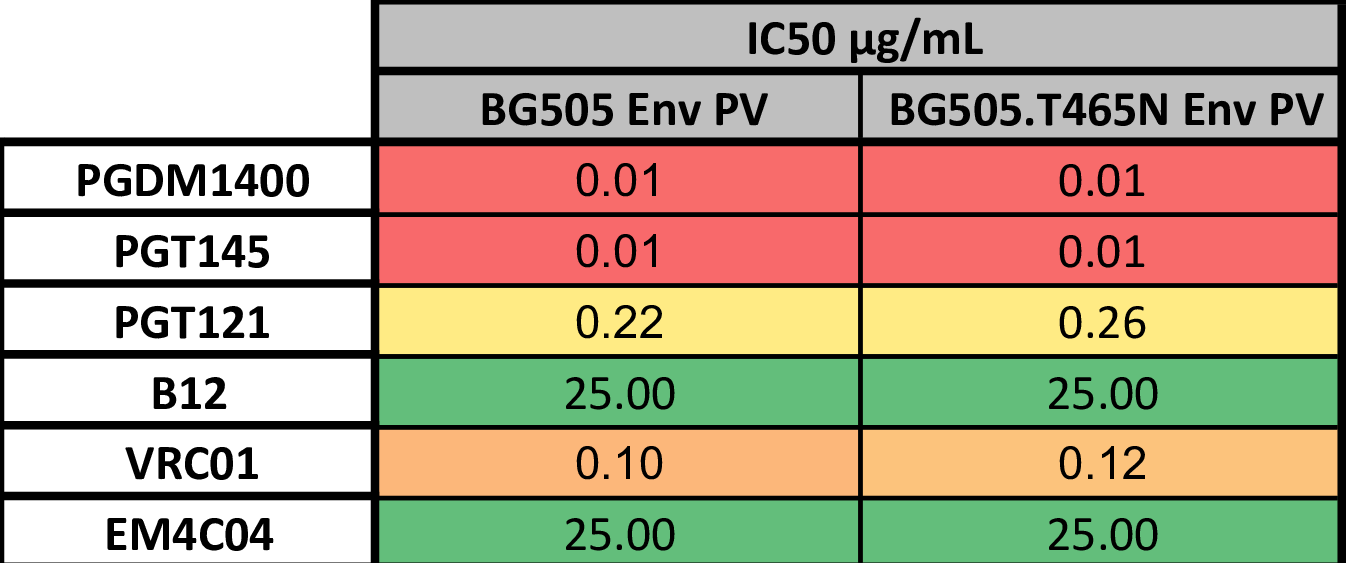

Supplement: S3 Fig — HIV broadly neutralizing antibodies were used to compare the sensitivity of the BG505.T465N mutant to BG505 Env PV in the TZM-bl assay. A heat map of the IC50 titers for each bnAb are shown in μg/ml, with 25 μg/ml being the highest concentration tested. The negative control anti-influenza HA mAb EM4C04 was included. Red to yellow indicates high to moderate susceptibility; yellow to green indicates resistance. (TIF) [file ppat.1009257.s003.tif]

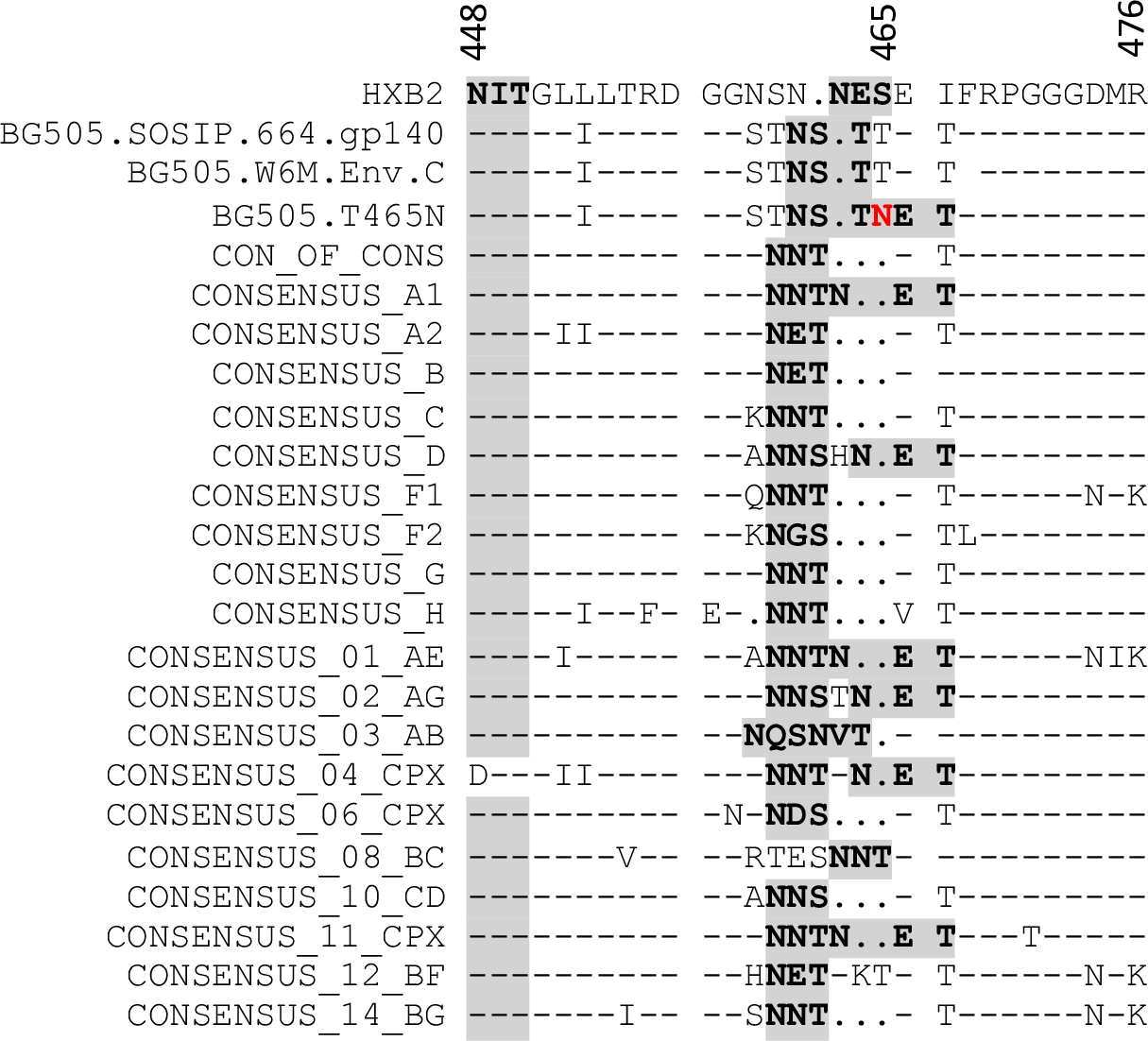

Supplement: S4 Fig — The amino acid sequence alignment contains BG505.SOSIP.664.gp140 (immunogen), BG505.W6M.Env.C (the parental PV), the BG505.T465N mutant, and a consensus for HIV-1 group M, all major HIV-1 clades and CRFs, with HXB2 as a reference sequence (Genbank K03455). The alignment was generated using https://www.hiv.lanl.gov/content/sequence/NEWALIGN/align.html and https://www.hiv.lanl.gov/content/sequence/SeqPublish/seqpublish.html. N-linked glycan motifs within this region are highlighted in gray and the T465N mutation is indicated in with red text. Dashes indicate conserved residues, while differences are shown, except within the 465 adjacent region, where glycan motifs are indicated by showing the amino acid residues (NXS/T where X is any residue except proline). Dots indicate a gap. (TIF) [file ppat.1009257.s004.tif]

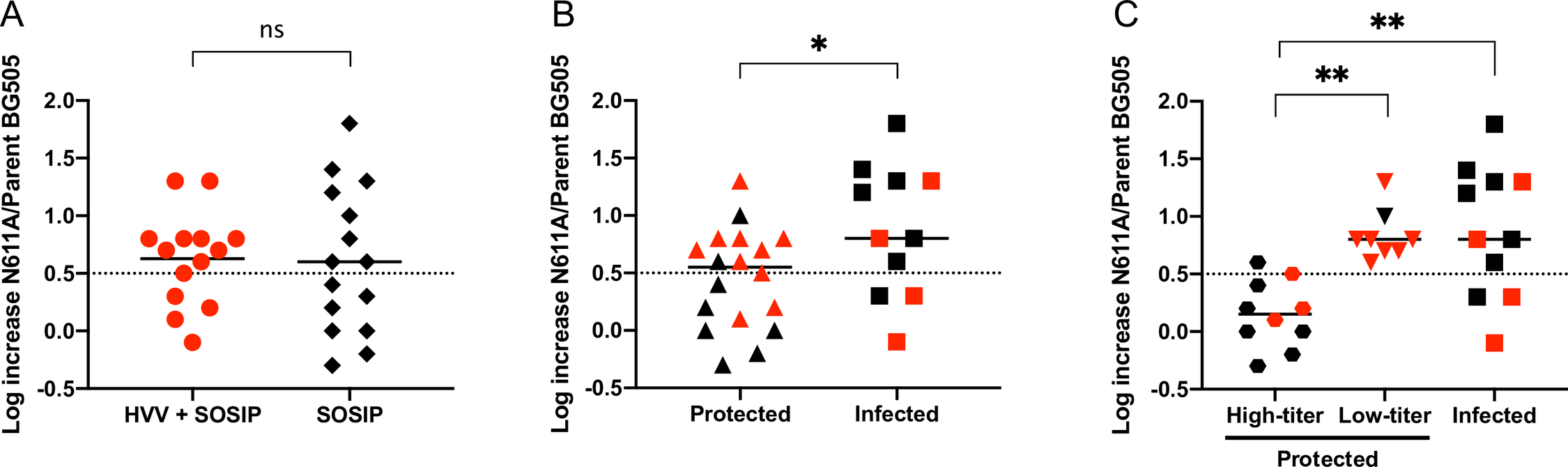

Supplement: S5 Fig — Serum neutralizing activity on the day of challenge (week 84) was evaluated against the parental BG505 and N611A mutant PVs using the TZM-bl assay for all but one immunized RM (n = 29). The log10 increase in ID50 titer of the N611A mutant compared to the parental BG505 Env is shown in vertical plots for each RM grouped by vaccination, challenge outcome, and titer/challenge outcome (A—C). Red symbols indicate HVV + SOSIP immunized RM; black symbols indicate SOSIP immunized RM. (A) vaccination groups HVV + SOSIP vs SOSIP (p>0.05), (B) protected vs infected (p = 0.047), and (C) high titer protected vs. low titer protected (p = 0.006) and low titer infected (p = 0.004) are shown. Mann-Whitney tests were used to perform the two group comparisons in (A) and (B) and a Kruskal-Wallis test with Dunn’s correction was used for the three-group comparison in (C). All were performed using GraphPad Prism (*p<0.05, **p<0.01). Horizontal bars in (A—C) represent the median of the group. The dashed lines indicate the threshold of 0.5log10 fold increase over the parental BG505. (TIF) [file ppat.1009257.s005.tif]

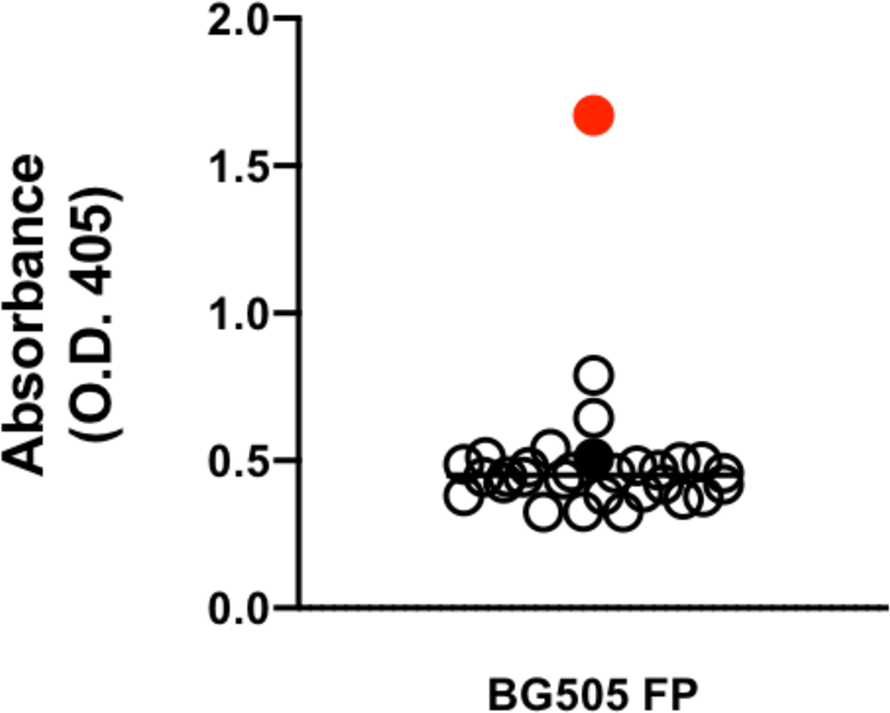

Supplement: S6 Fig — BG505 fusion peptide specific IgG was measured by ELISA on the day of challenge (week 84) using serum from 30 immunized RM. His-tagged fusion peptide was captured onto the plate wells. Each ELISA was run with duplicate wells and repeated independently at least twice. The horizontal bars indicates the median. Filled symbols indicate the positive control, bnAb VRC34 (red), and negative control anti-influenza HA mAb, EM4C04 (black). (TIF) [file ppat.1009257.s006.tif]

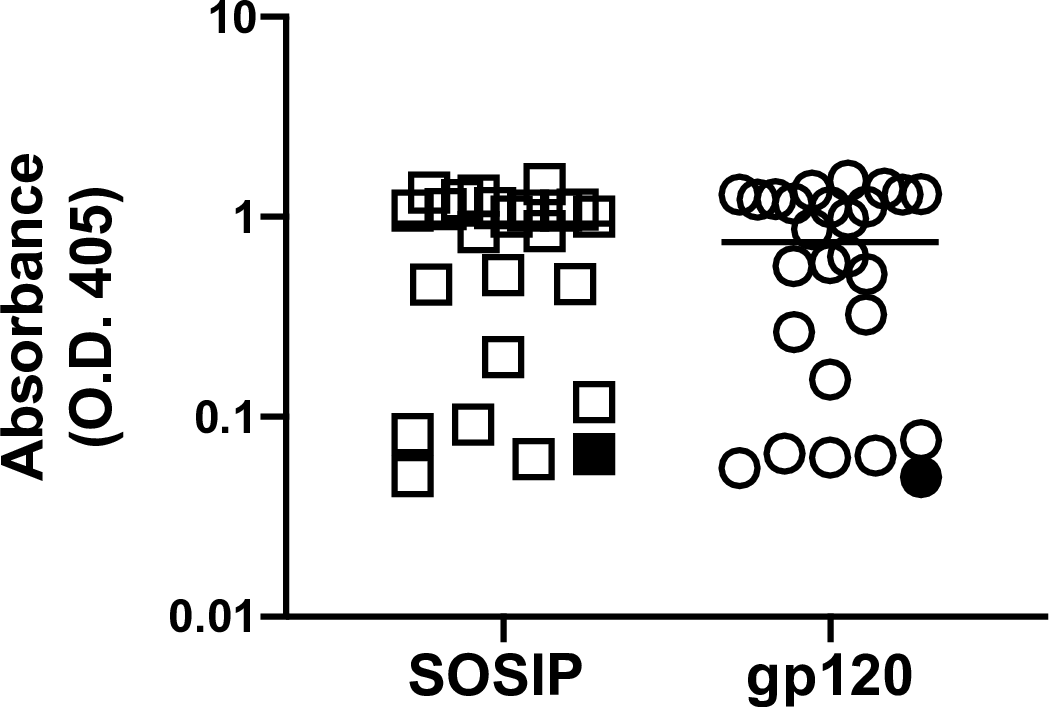

Supplement: S7 Fig — An ELISA was performed using 18 SOSIP-sorted and 20 gp120-sorted mAbs to measure binding to BG505 SOSIP, which was captured onto the plate wells. The absorbance was measured using each mAb at 8 μg/ml. Each ELISA was run with duplicate wells and repeated independently at least twice. Horizontal bars indicate the median. Filled black symbols indicate the negative control anti-influenza HA mAb, EM4C04. (TIF) [file ppat.1009257.s007.tif]

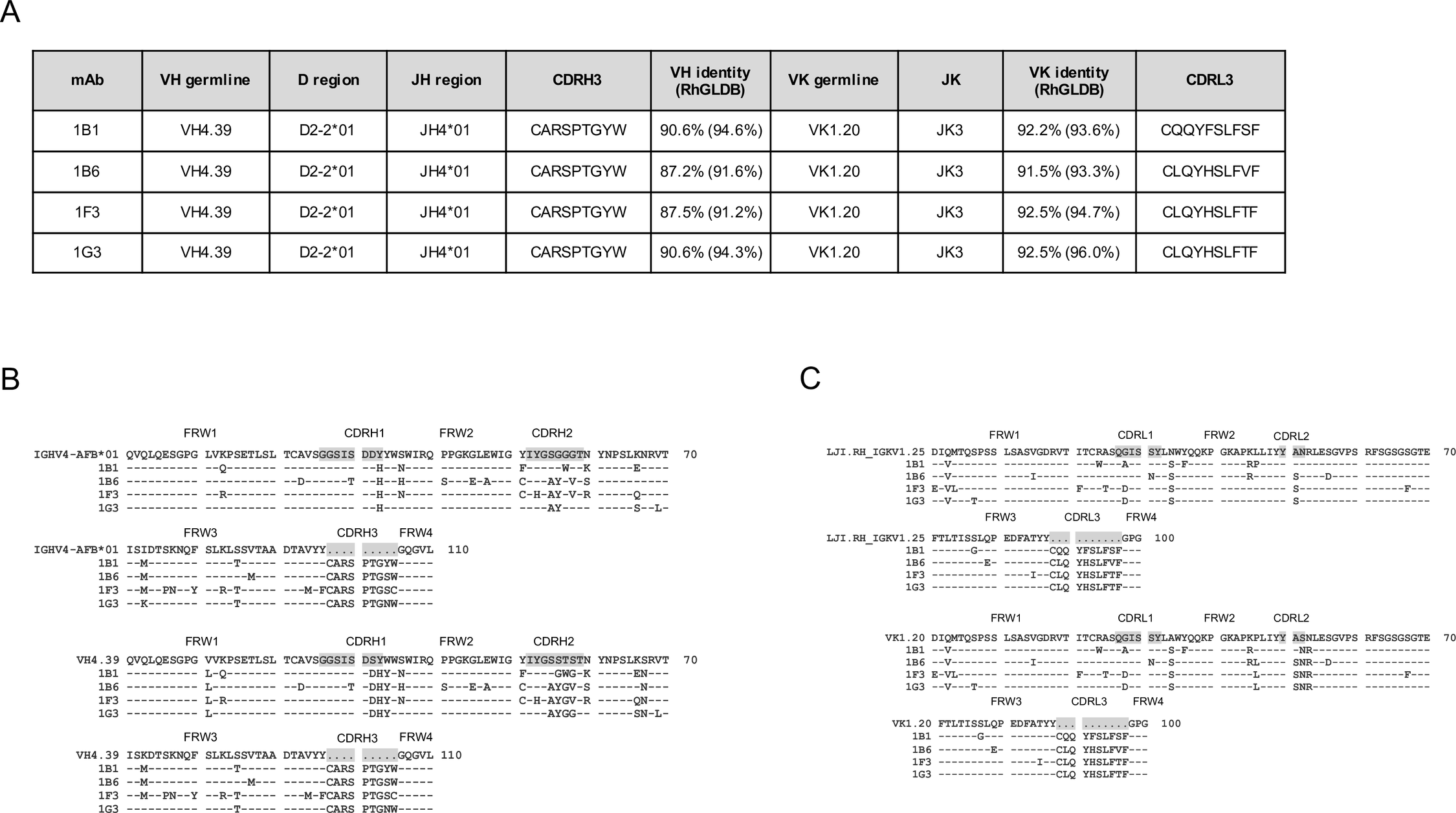

Supplement: S8 Fig — The nucleotide sequences of the variable domains of the four neutralizing mAbs were submitted to IgBlast, the VDJ and VJ assignments for the heavy and light chain, respectively, are shown in (A) The nucleotide identity to the assigned germline from IgBlast, and from a second database RhGLDB in parenthesis, is also shown for each mAb. Amino acid alignments of the four heavy chains with putative germlines, IGHV4-AFB*01 (RhGLDB) and VH4.39 (IgBLAST) in (B) and the four light chains with putative germlines, LJI.RH_IGKV1.25 (RhGLDB) and VK1.20 (IgBLAST) in (C) are shown. Identity with the germline is indicated by a dash, and amino acid differences are shown. The framework (FRW) and complementarity determining regions (CDR, shaded) are indicated above the sequences. Dots indicate that the sequence was not present in the germline (CDRH3 and CDRL3). (TIF) [file ppat.1009257.s008.tif]

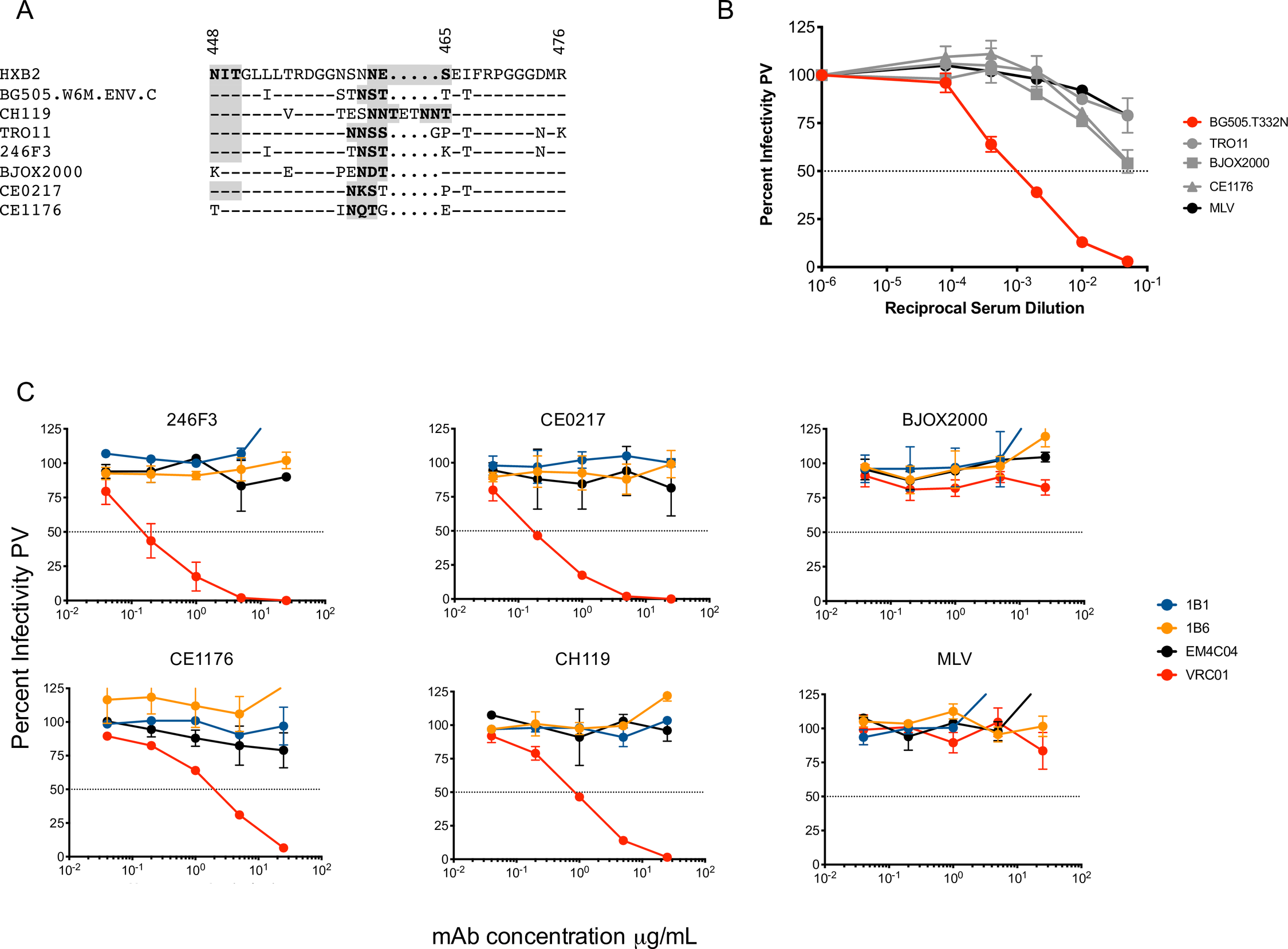

Supplement: S9 Fig — The sequence alignment of heterologous HIV-1 Envs proximal to residue 465 are shown in (A). The amino acid sequence alignment contains BG505.W6M.Env.C (DQ208458, clade A), and HIV-1 Envs CH119 (EF117271, CRF07), TRO11 (AY835445, clade B), 246F3 (HM215279, clade AC), BJOX2000 (HM215364), CRF07), CE0217 (FJ443575, clade C), CE1176 (FJ444437, clade C), with HXB2 (K03455) shown as a reference sequence. The alignment was generated using Geneious v9. N-linked glycan motifs within this region are highlighted in gray. Dashes indicate conserved residues, while differences are shown, except within the 465 adjacent region, where glycan motifs are indicated by showing the amino acid residues (NXS/T where X is any residue except proline). Dots indicate a gap in the alignment. The TZM-bl assay was used to assess neutralizing activity against the panel of PVs using serum (B) and mAbs (C) from RUp16 following the third protein immunization. (B) A key indicating the Envs used to evaluate serum neutralizing activity is shown to right of the graph. The reciprocal of the serum dilution is plotted on the x axis on a log10 scale and the percent of viral infectivity is plotted on the y axis relative to the virus only control at 100%. (C) Neutralizing mAbs isolated from RUp16 were tested against the Envs indicated above each graph. The percent of viral infectivity is plotted against mAb concentration in μg/ml. For comparison, murine leukemia virus (MLV) Env was included as a negative control (B and C). mAbs VRC01 and EM4C04 were used as positive and negative controls, respectively. (TIF) [file ppat.1009257.s009.tif]

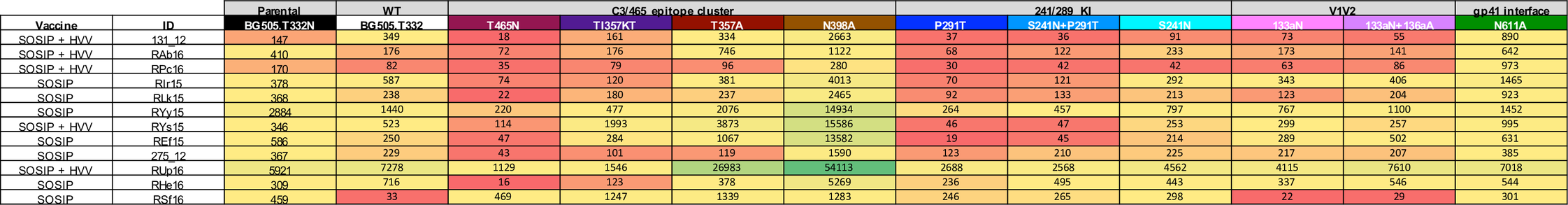

Supplement: S1 Table — (TIF) [file ppat.1009257.s010.tif]

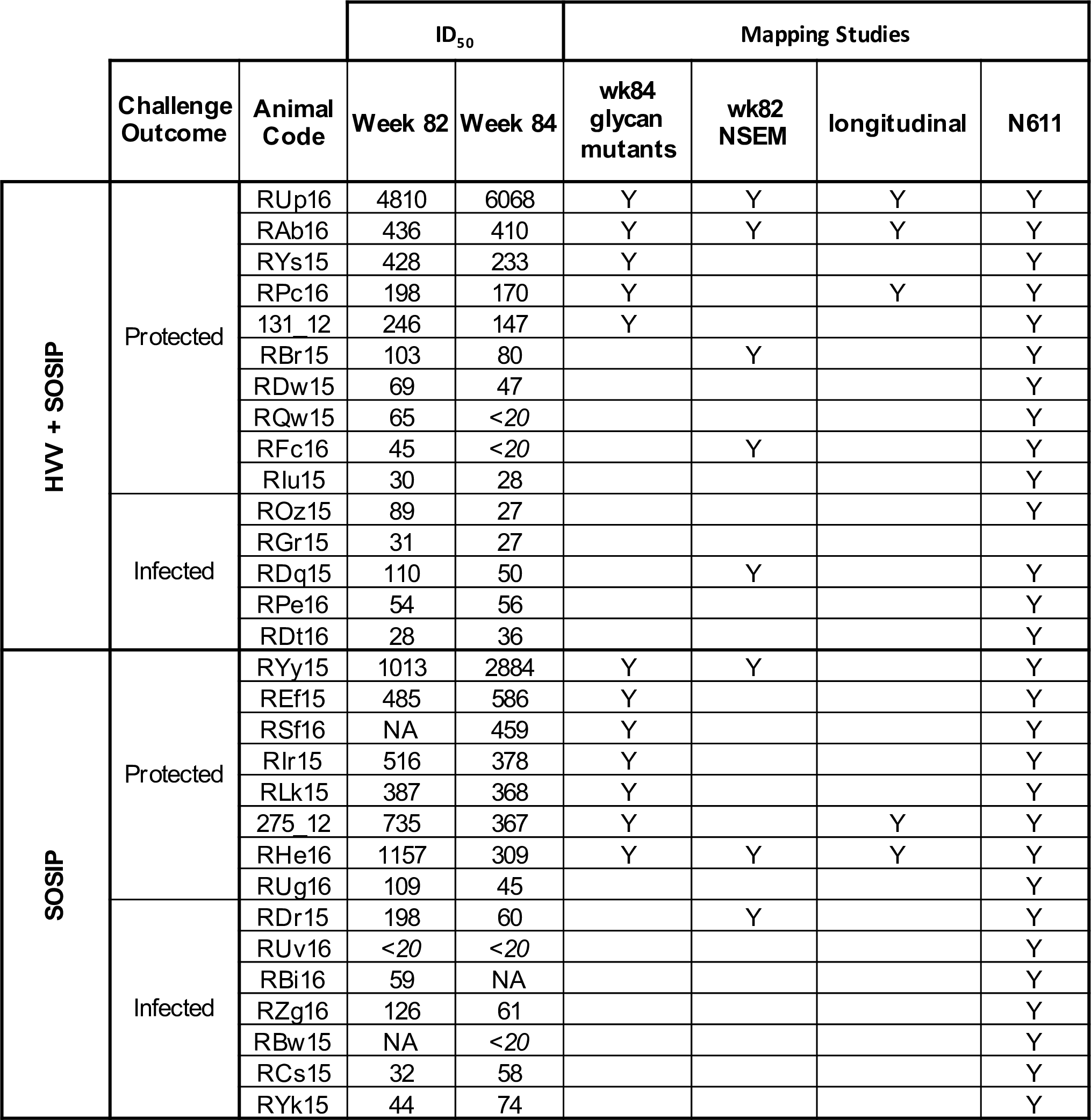

Supplement: S2 Table — (TIF) [file ppat.1009257.s011.tif]
